# Attenuated Bacteria‐Based Tumor Therapy: Clinical Application Risks, Marketing Approval Restrictions, and Coping Strategies

**DOI:** 10.1002/advs.202517730

**Published:** 2026-02-16

**Authors:** Yucheng Liu, Junhong Yao, Jiaxin Deng, Minjie Zhou, Kaiyue Shen, Shishuo Sun, Xiaoge Gao, Qing Zhang, Haiheng Xu

**Affiliations:** ^1^ Cancer Institute, Cellular Therapeutics School of Medicine Xuzhou Medical University 209 Tongshan Road Xuzhou Jiangsu China; ^2^ Center of Clinical Oncology The Affiliated Hospital of Xuzhou Medical University Xuzhou Jiangsu China; ^3^ Jiangsu Center for the Collaboration and Innovation of Cancer Biotherapy Xuzhou Medical University Xuzhou Jiangsu China; ^4^ Laboratory Animal Center Nanjing University of Chinese Medicine Nanjing China

**Keywords:** attenuated bacteria, bacterial products, cancer immunotherapies, drug delivery systems

## Abstract

Bacteria‐based cancer therapy is a promising cancer treatment strategy that utilizes genetically engineered attenuated bacteria to specifically target tumor tissues, directly kill cancer cells, or activate the host's immune response. Engineering modifications of attenuated bacteria can achieve more effective anti‐tumor effects, and consequently, a number of clinical trials have been carried out. However, the clinical translation of attenuated bacteria faces multiple challenges, including safety risks, fluctuating therapeutic efficacy, difficulties in vivo monitoring, complex production quality control, and lack of regulatory approval standards. This review mainly summarizes five aspects of bacterial cancer therapy: the safety risks and attenuation strategies, the regulation of therapeutic stability, bacterial in vivo imaging technologies, the optimization of bacterial production processes, and market approval pathways. It aims to precisely control bacterial behavior through genetic circuits and enable real‐time monitoring of the treatment process by means of multimodal imaging, ultimately promoting the clinical translation of attenuated bacterial therapy.

## Graphical abstract

This review comprehensively overviews attenuated bacteria‐based tumor therapy, highlighting key methodologies and recent progress. It examines strategies for enhancing safety, stabilizing efficacy, optimizing manufacturing, and navigating regulatory pathways. We aim to guide scientists and microbiologists in engineering optimal attenuated bacteria, while aiding in overcoming current obstacles to accelerate the clinical translation of this therapeutic approach.

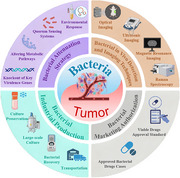

## 1 Introduction

Since William Coley's pioneering application of bacterial extracts (Coley toxins) for tumor treatment in the late 19th century, attenuated bacterial therapy has evolved from empirical exploration to rational design [1]. Modern attenuated bacterial therapy employs synthetic biology techniques to precisely engineer bacteria for tumor‐targeted colonization [2], inducing antitumor effects via toxin secretion or immune activation [3, 4]. Attenuated bacteria exhibit unique advantages over conventional therapies, including penetration of dense tumor stroma, survival in hypoxic and acidic microenvironments, and sustained local drug delivery [5, 6]. Furthermore, engineered bacteria can remodel the tumor immune microenvironment by reversing immunosuppression and enhancing T cell‐mediated tumor recognition and elimination [7]. Currently, commercialized bacterial drugs and ongoing clinical trials utilizing attenuated bacteria highlight the therapeutic potential and progress in tumor treatment [8].

**FIGURE 1:**
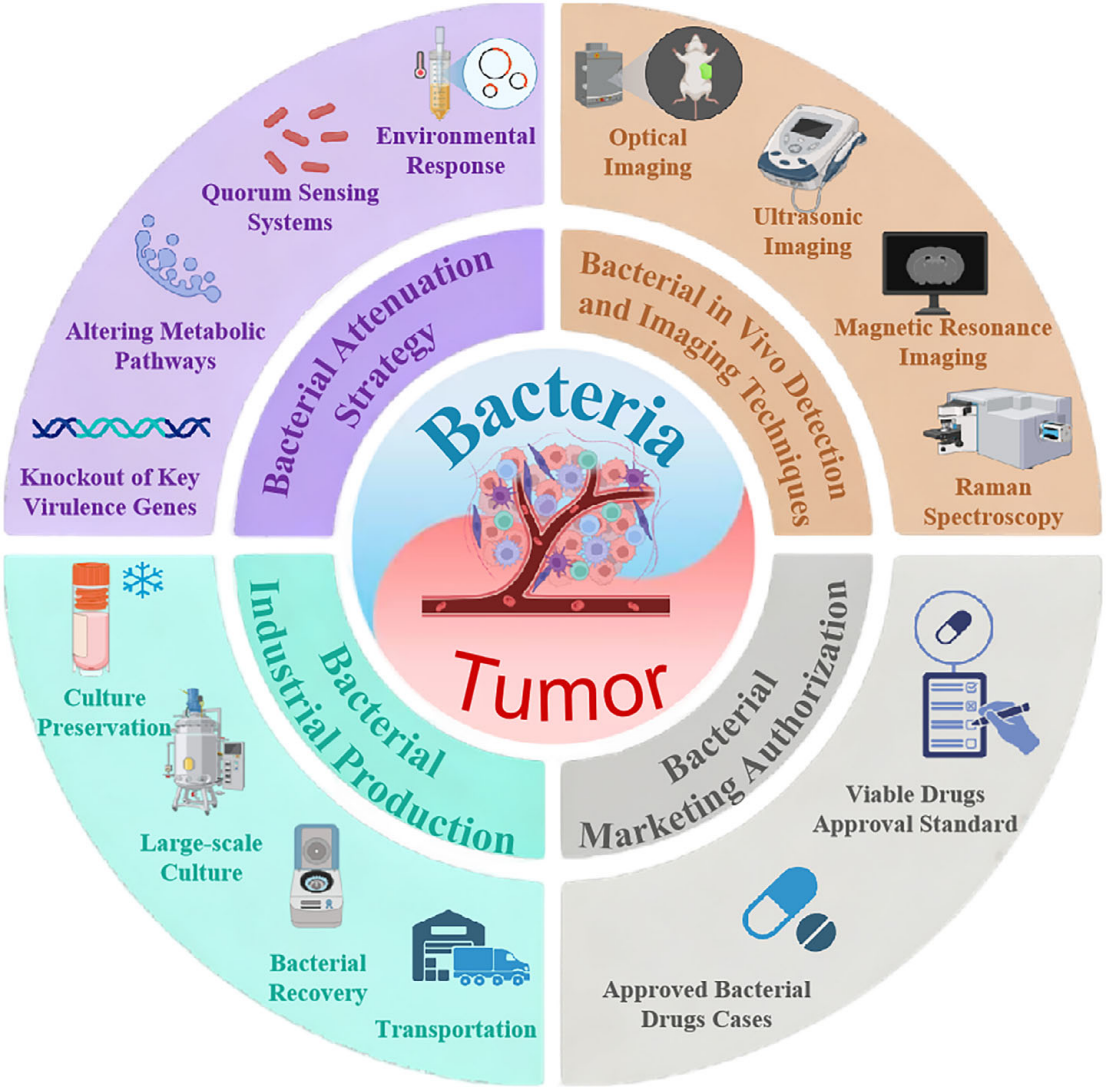
An overview of attenuated bacteria‐based tumor therapy, including the bacterial attenuation strategy, the bacterial in vivo detection and imaging techniques, the bacterial industrial production, and the bacterial marketing authorization. Created with Biorender.com.

Despite its therapeutic promise, attenuated bacterial therapy faces significant challenges in clinical translation. Safety concerns include excessive immune activation and off‐target bacterial dissemination, which may cause systemic infections or hyperinflammatory responses [9]. Therapeutic efficacy is compromised by dynamic fluctuations in bacterial proliferation, distribution, and metabolism in vivo, making precise spatiotemporal control of treatment effects difficult [10]. Manufacturing challenges include underdeveloped standardized protocols for large‐scale cultivation and quality control, while inherent biological complexity causes batch‐to‐batch variability. Furthermore, dedicated regulatory frameworks remain exploratory owing to the unique biological nature of these therapeutics. To overcome these limitations, we systematically examine strategies for improving safety profiles, enhancing efficacy consistency, optimizing manufacturing, and navigating regulatory pathways. We propose integrating attenuation strategies with viability monitoring to establish a closed‐loop ‘design‐monitor‐regulate’ system. This system would enable genetic circuit‐based control of bacterial behavior coupled with multimodal imaging for real‐time therapeutic monitoring. Collectively, these advancements could facilitate the safe clinical implementation of attenuated bacterial therapies (Figure 1).

## 2 Clinical Application Safety Risks and Attenuation Strategies

Despite their application in cancer therapy, attenuated bacteria still pose significant safety challenges [11]. Sustained bacterial colonization and metabolic activity in healthy tissues may trigger chronic inflammation through endotoxin/exotoxin release, potentially causing tissue damage and progressive organ dysfunction. Therefore, developing reliable methods to concurrently reduce bacterial toxicity, minimize immune‐related adverse events, and improve targeting precision has become crucial for advancing attenuated bacterial therapies.

### 2.1 Traditional Attenuation Strategies

Traditional attenuation approaches (e.g., serial passage or random mutagenesis) carry inherent risks of virulence reversion owing to their empirical design. The VNP20009 Salmonella strain exemplifies this limitation: while demonstrating preclinical antitumor efficacy, it caused persistent colonization in healthy organs (particularly liver and spleen), leading to clinical trial termination [5, 12]. Furthermore, ectopic colonization and systemic dissemination present additional safety concerns. Excessive immune activation may trigger systemic inflammation, with CRS constituting a particularly lethal barrier to clinical translation. These limitations collectively hinder the safe clinical implementation of attenuated bacterial therapies.

#### 2.1.1 Continuous Passage Culture

Serial passage culture represents a classical attenuation approach, wherein bacteria are repeatedly subcultured under controlled laboratory conditions. This method relies on forced bacterial adaptation to artificial growth conditions, leading to progressive loss of virulence factors through successive generations. However, this evolution‐driven attenuation strategy has significant limitations: First, the process requires extensive time (often years) to achieve stable attenuation. Second, the unclear molecular basis of attenuation risks virulence reversion and complicates in vivo behavior control. Third, random mutations may disrupt metabolic or adhesive properties, compromising tumor targeting and precision therapeutic potential.

The early application of serial subculture for attenuation dates back to the development of *Bacillus Calmette‐Guérin* (BCG), which was derived from serial subculturing of *Mycobacterium bovis*. Its core mechanism relies on forced adaptation within artificial media (glycerin‐bile‐potato medium), which leads to the stochastic loss of non‐essential virulence factors, such as secreted toxins or surface adhesins. In nutrient‐rich environments devoid of immune pressure, maintaining these virulence factors imposes a significant metabolic burden without conferring any selective advantage. Consequently, mutant sub‐populations that spontaneously lose virulence genes exhibit superior growth kinetics and eventually outcompete virulent strains [13].

**FIGURE 2:**
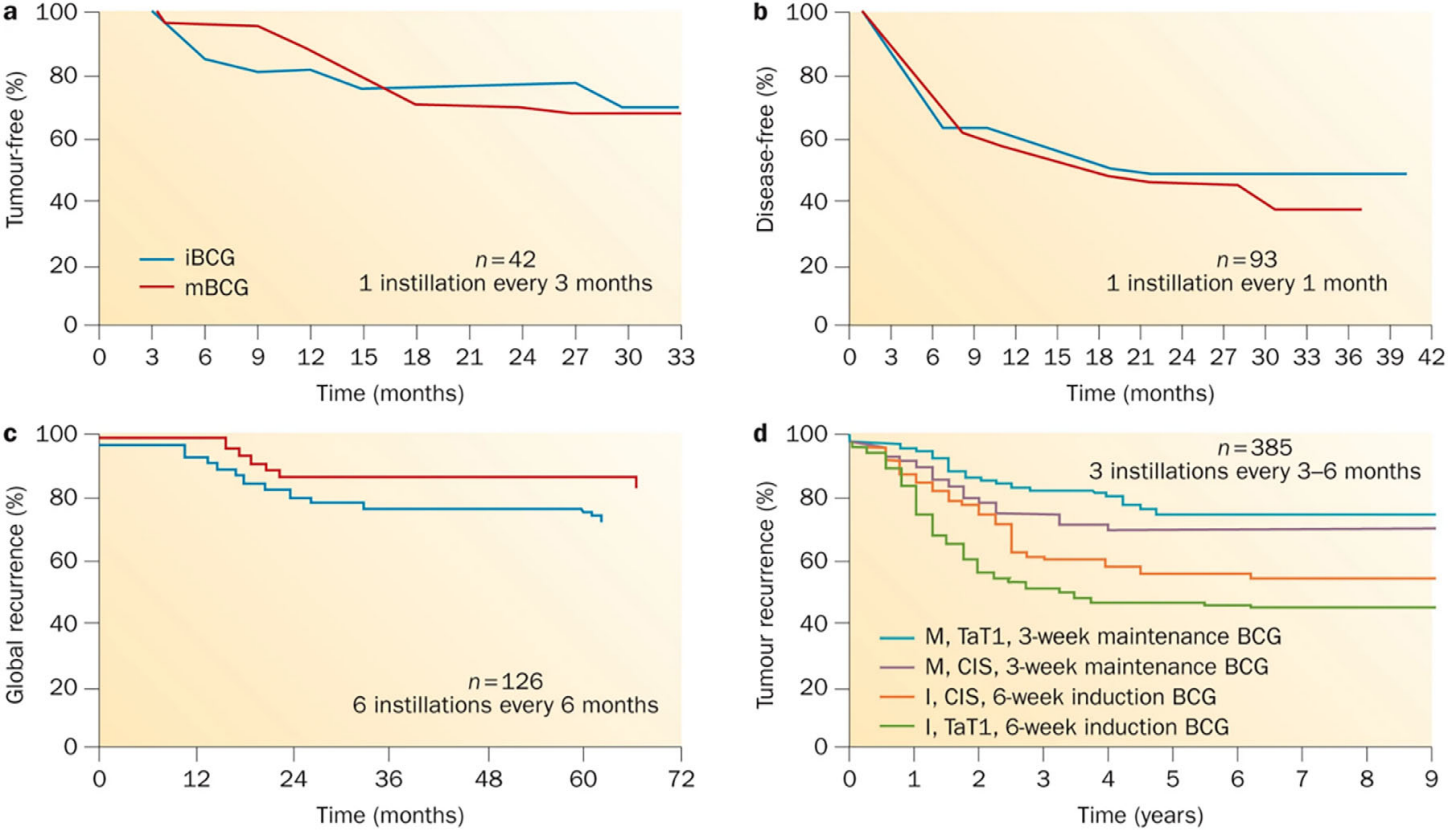
Efficacy of BCG therapy varies by maintenance schedule [14]. (a–c) Kaplan–Meier curves demonstrate that recurrence trends in three clinical trials show no difference between BCG induction alone compared to BCG maintenance (suboptimal schedule). (d) Kaplan–Meier curves reflect the importance of true maintenance therapy on tumour recurrence for superficial bladder cancer of all stages. Reproduced with permission [14]. Copyright 2015, Springer Nature.

Clinical studies by Kamat et al. in the 1970s confirmed BCG's efficacy against superficial bladder cancer, establishing it as the first viable bacterial preparation approved for bladder cancer treatment and laying a foundational basis for the clinical translation of bacterial therapies (Figure 2) [14].

Although continuous subculture has been applied in bacterial attenuation, its inherent limitations—such as the prolonged timeline required for attenuation, ambiguities in virulence mechanisms, risks of toxicity reversion, and unintended effects on bacterial traits—necessitate supplementation or replacement with more advanced and precise technologies.

#### 2.1.2 Chemical or Physical Mutagenesis

Traditional attenuation methods include chemical (e.g., nitrous acid, EMS) and physical mutagens (e.g., UV, γ‐irradiation), which induce random genomic mutations to reduce virulence [15]. The primary principle involves the induction of genomic lesions: ultraviolet (UV) radiation promotes the formation of pyrimidine dimers, leading to replication errors, while alkylating agents such as N‐methyl‐N'‐nitro‐N‐nitrosoguanidine (NTG) facilitate transition mutations from G‐C to A‐T base pairs.

Ji et al. irradiated Salmonella typhimurium strain ST454 with 1.5 kGy gamma rays, followed by screening via in vitro macrophage replication assays and in vivo mouse infection tests, yielding the attenuated strain ATOM Sal‐L6. This strain exhibited a 9 961‐fold reduction in virulence relative to the wild‐type strain and could induce protective immune responses against homologous pathogenic infections in mice. In mouse models, intramuscular or oral vaccination with ATOM Sal‐L6 elicited Salmonella‐specific IgG antibodies and multifunctional T cell responses, with 60% of mice surviving challenge with the wild‐type strain [16].

Due to the randomness of chemical and physical mutagenesis, mutation outcomes remain unpredictable. Bacterial functions—including chemotaxis, motility, and nutrient sensing—are typically regulated by intricate polygenic networks. Random mutagenesis indiscriminately dismantles these complex regulatory frameworks, as the disruption of any critical component, such as chemotactic receptors, flagellar motor proteins, or metabolic sensors, can lead to the unpredictable attenuation or complete loss of these functions. Consequently, although these methods achieve attenuation by disrupting bacterial virulence genes, they also pose a high risk of adversely affecting key tumor‐targeting functions, such as bacterial chemotaxis and metabolic enzyme activity [15].

The inability to precisely control mutation sites complicates the safety evaluation of mutagenized attenuated strains: this process not only relies heavily on long‐term in vivo observation, but the persistent risk of off‐target effects also necessitates extensive screening to identify safe and effective attenuated strains. Thus, despite their feasibility, chemical and physical mutagenesis encounter significant challenges in practical application, arising from uncertainties in mutation outcomes, complexities in safety assessment, and the burdensome screening requirements they impose.

### 2.2 Attenuation Strategies of Engineered Bacteria

Traditional attenuation methods are plagued by safety concerns, including the risk of toxicity reversion, hazards of off‐target dissemination, and immune overactivation, all of which severely impede their translation from basic research to clinical application. The lack of specificity in passage attenuation and the inherent randomness of chemical/physical mutagenesis not only hinder precise control of bacterial virulence but also risk disrupting critical bacterial functions. This duality creates a persistent dilemma: achieving a balance between therapeutic efficacy and safety remains elusive, as efforts to reduce toxicity may inadvertently compromise key traits such as tumor targeting or immune‐modulating capacity, while preserving functionality often leaves residual safety risks unaddressed. These limitations highlight the need for more rational and controllable approaches to bacterial attenuation.

Engineered bacterial attenuation strategies, which rely on precise manipulation of gene‐editing technologies, provide a solution to the inherent limitations of traditional methods. By directionally modifying bacterial virulence genes, regulating survival‐dependent conditions, or introducing responsive switches, these strategies offer a novel approach to preventing toxicity reversion, restricting off‐target colonization, and modulating immune responses.

#### 2.2.1 Knockout of Key Virulence Genes

Advances in molecular biology techniques have enabled genetic engineering to open more precise avenues for bacterial attenuation. The knockout of key virulence genes involves the specific deletion, via gene‐editing technologies, of bacterial genes closely associated with pathogenicity, thereby reducing bacterial virulence. Compared with traditional methods relying on random mutagenesis or passage attenuation, this approach not only significantly reduces the risk of toxicity reversion but also confers specific responsiveness to bacteria within the tumor microenvironment, thereby achieving simultaneous improvements in safety and efficacy.

VNP20009 is one of the earliest attenuated Salmonella strains to enter clinical trials for tumor‐targeted therapy. Derived from wild‐type *Salmonella Typhimurium* 14028s, this strain features the excision of *purI* to establish purine auxotrophy, rendering bacterial proliferation dependent on the purine‐rich environment of tumor tissues for targeted colonization; concurrently, the deletion of *msbB* modifies the Lipid A structure to reduce endotoxicity and mitigate systemic inflammatory responses [17].

Building upon the dual‐gene deletion framework of VNP20009, subsequent studies have further expanded the repertoire of genetic targets to address multifaceted aspects of bacterial pathogenicity, thereby enabling the continued optimization of the safety and efficacy profiles of attenuated bacteria. The *htrA* gene encodes a periplasmic serine protease that exhibits dual molecular chaperone and proteolytic activities. It primarily maintains bacterial outer membrane proteostasis and stress tolerance by degrading misfolded or damaged periplasmic proteins under stress conditions, thereby facilitating bacterial survival within macrophages. Wu et al. utilized the CRISPR‐Cas9 system [18] to knockout the *htrA* gene in VNP20009, constructing the attenuated Salmonella strain ΔhtrA‐VNP (designated AIS). This modification reduced EPS content, weakened bacterial resistance to macrophage‐mediated clearance, and significantly decreased toxicity, while preserving the strain's ability to colonize and proliferate within tumors [12].

**FIGURE 3:**
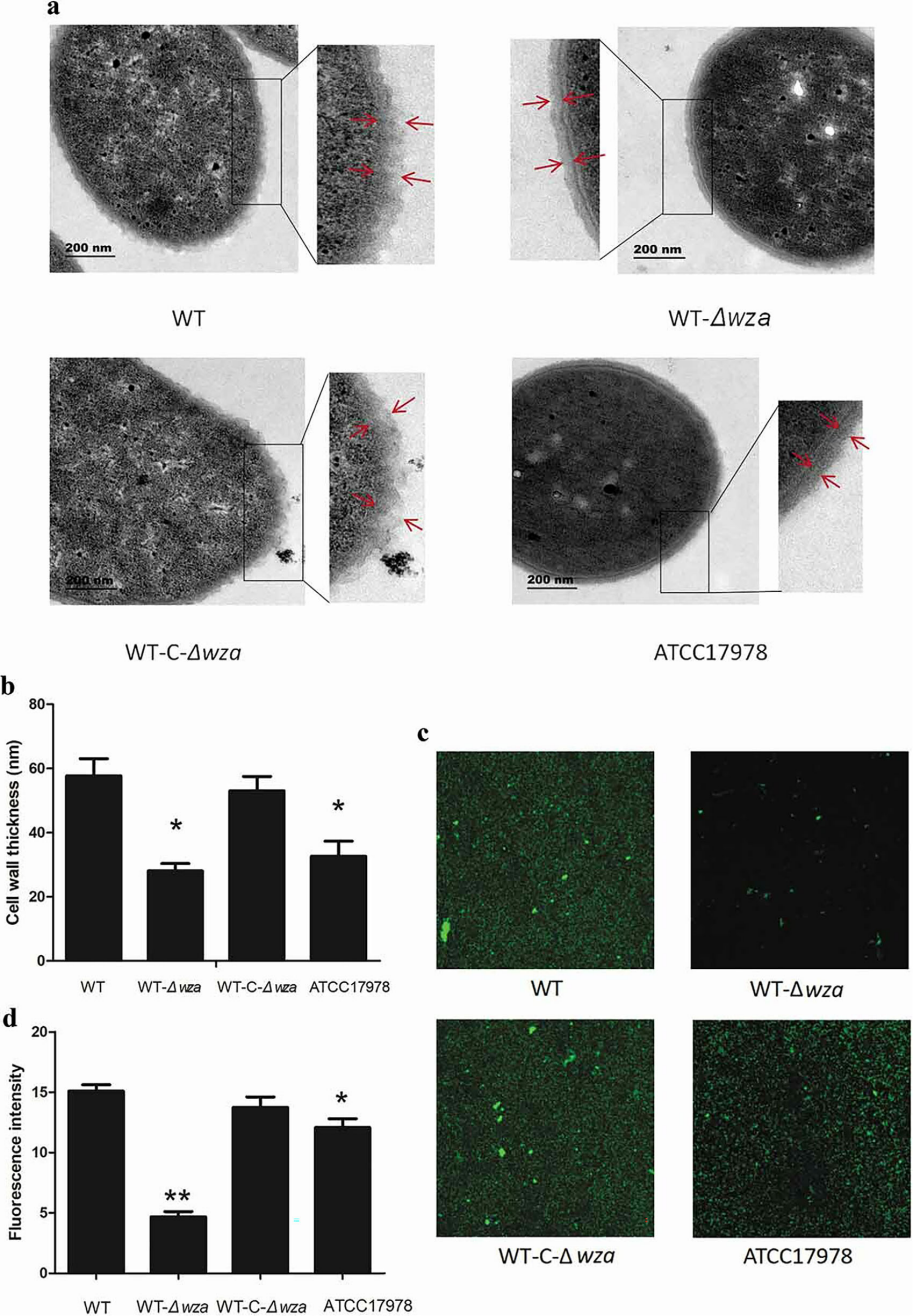
Bacterial morphology and biofilm formation [19]. (a) Observation of bacterial capsules by transmission electron microscopy (9800×). The red tip shows the cell wall of bacteria. (b) Statistical analysis of the thickness of bacterial cell wall. (c) Laser scanning Confocal Microscopy to observe biofilm formation. Green fluorescence shows the biofilm of bacteria. The intensity of green fluorescence reflects the thickness of the biofilm. (d) Statistical analysis of the intensity of green fluorescence. WT is wild type strain, WT‐Δwza is *wza* knockout strain, WT‐C‐Δwza is *wza* replenishment strain, and ATCC17978 is control strain Error bars indicate the s.d. for the three biological replicates examined for each strain. Asterisks indicate statistically significantly different from SKLX024256 WT (*P < 0.05, **P < 0.01, one‐way unpaired analysis of variance, *n* =  3). Reproduced with permission [19]. Copyright 2020, Taylor & Francis.

Beyond Salmonella‐based models, targeted genetic modifications have been successfully applied to other therapeutic bacteria to modulate their structural virulence factors. In *Acinetobacter baumannii* (AB), the *wza* gene encodes an outer membrane auxiliary protein (Wza), which forms an octameric channel responsible for the trans‐outer membrane transport and surface anchoring of polymerized capsular polysaccharides (CPS). An intact capsule layer serves as the primary physical barrier protecting bacteria against host complement‐mediated killing, macrophage phagocytosis, and oxidative stress, while also promoting surface adhesion and biofilm formation. Niu et al. constructed a suicide plasmid to knockout *wza*, thereby blocking AB capsular polysaccharide synthesis; this resulted in a significant reduction in cell wall thickness (to 32.26 nm, compared with 61.48 nm in the wild‐type strain), leading to decreased bacterial virulence (Figure 3) [19].

Through precise targeting of specific genes, key virulence gene knockout technology allows for the deletion of critical pathogenic determinants, such as toxin‐encoding genes or host adhesion‐related genes. This approach overcomes the inherent limitations of traditional attenuation methods, such as incomplete or unstable virulence reduction, while avoiding unnecessary disruption of other essential bacterial physiological functions. Consequently, attenuated bacteria exhibit significant improvements in both safety and efficacy.

#### 2.2.2 Altering Metabolic Pathways

Metabolites can function as signaling molecules to regulate the expression of virulence genes, rendering the modification of bacterial metabolic pathways a key strategy for modulating bacterial virulence and expanding bacterial functions.

The first is auxotrophic modification, which involves deleting genes critical for bacteria to synthesize essential nutrients (e.g., amino acids), thereby restricting their survival to tumor sites with specific nutrient supplementation. For instance, the hypermethylation of histone H3 observed across various sarcoma PDX models is dependent on tumor‐supplied methionine as a methyl donor. This provides a molecular rationale for the design of methionine‐dependent auxotrophic attenuated bacteria [20].

Besides, deletion of the *purI* gene in VNP20009 impairs its ability to synthesize purines, confining its proliferation to purine‐rich tumor microenvironments while rendering it barely viable in normal tissues [17]. Similarly, the attenuated Listeria strain ΔprfA depends on glutamine and glutathione present in the tumor microenvironment to activate its virulence genes, which significantly reduces the risk of off‐target colonization [21, 22, 23]. Zhao et al. initially generated the leucine‐arginine auxotrophic attenuated strain A1 through NTG mutagenesis, followed by the selection of the enhanced strain A1‐R via serial passage through tumors. This strain exhibits exceptional tumor‐targeting specificity and antitumor potency, enabling robust proliferation within the tumor while growth remains strictly restricted in normal tissues due to nutritional constraints, thereby ensuring the safety profile of A1‐R. In subsequent studies, Zhao et al. demonstrated that A1‐R successfully eradicated tumors in orthotopic breast cancer models. Furthermore, systemic administration of A1‐R effectively cleared both primary orthotopic lesions and distant metastases in prostate cancer, ultimately achieving complete tumor regression in the majority of treated mice. This body of research confirms that amino acid auxotrophic bacteria with selective tumor colonization capabilities hold significant promise for oncological therapeutics [24, 25, 26].

Metabolic pathway modification influences bacterial virulence by redesigning and optimizing bacterial metabolic networks, thereby altering their metabolic processes and product synthesis. Under environmental stressors—such as nutritional fluctuations or oxidative stress—ppGpp in wild‐type bacteria binds to RNA polymerase to execute a dual regulatory response: it downregulates the synthesis of rRNA and tRNA to arrest cell division and minimize basal metabolic consumption, while simultaneously activating key factors like RpoS (the master regulator of stress survival) to trigger the secretion of virulence factors and the expression of stress‐resistance genes. Conversely, the deletion of ppGpp synthetase genes compels the bacteria to maintain high‐energy growth blindly under intratumoral stress, ultimately leading to metabolic exhaustion. Furthermore, the inability to initiate essential defense and virulence programs renders these strains highly susceptible to host clearance, thereby achieving a stable attenuated phenotype.

This metabolic deficiency does not diminish the immunogenicity of the bacteria, the ΔppGpp strain exhibits negligible toxicity in mice while retaining the ability to induce robust systemic and mucosal immune responses. This indicates an effective balance between safety and immune activation [27]. Sun et al. constructed the HCS1 strain by knocking out the ppGpp synthase genes *relA* and *spoT* in Salmonella VNP20009. HCS1 was found to increase the levels of inflammatory cytokines (e.g., TNF‐α and IL‐1β) in tumor tissues, promote the recruitment of immune cells to tumor sites, and decrease the level of the anti‐inflammatory cytokine IL‐10—thereby effectively alleviating the immunosuppressive state of the tumor microenvironment and enhancing immune activation [28].

Metabolic pathway modification not only exerts significant effects in bacterial attenuation but also endows bacteria with novel functional traits, providing possibilities for treating various diseases and developing new therapeutic approaches.

#### 2.2.3 Quorum Sensing Systems

**FIGURE 4:**
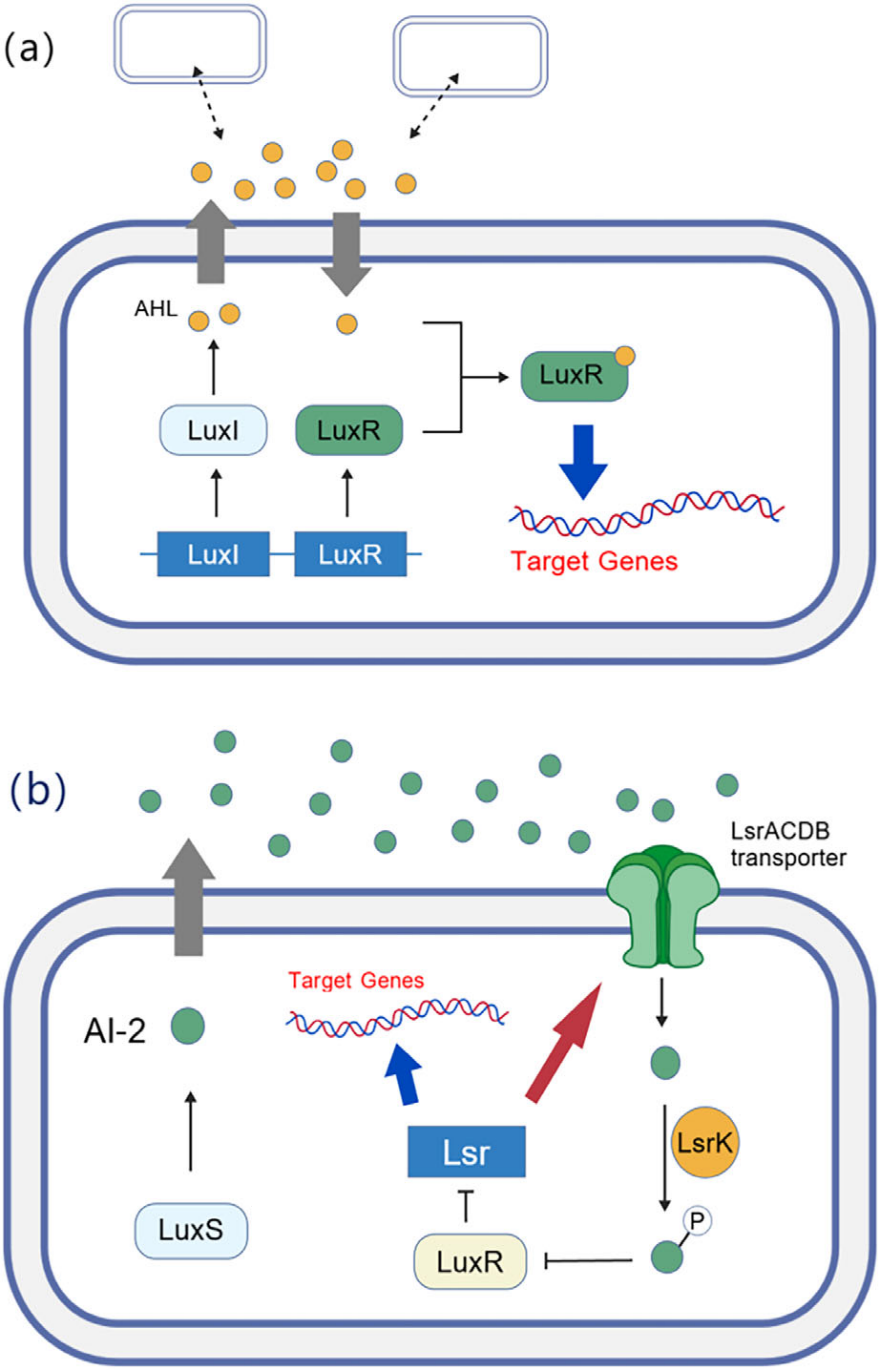
(a) Acylhomoserine lactone (AHL) system. LuxI produces the quorum sensing factor AHL, which is released into the environment. When its concentration reaches a threshold, AHL binds to the LuxR protein to form a complex, which initiates the expression of related genes (e.g., virulence factor genes and biofilm‐associated genes). (b) Furanoylboronic acid diester (AI‐2) system. AI‐2 enters the cell via the transporter LsrACDB; after being phosphorylated by LsrK, it relieves the inhibition of LsrR on the Lsr operon, activates other functional genes, and induces the cell to produce more AI transporters, thereby forming a positive feedback loop.

Quorum sensing (QS) is a gene regulatory mechanism by which bacteria coordinate collective behaviors through the secretion, sensing, and response to specific signaling molecules. The core of QS lies in the synchronous regulation of group behaviors (e.g., biofilm formation, virulence factor expression, antibiotic synthesis, and collective motility) when the concentration of signaling molecules reaches a threshold as population density increases.The primary mechanisms are illustrated in Figure 4.

Wu et al. constructed the AISI‐H engineered strain by inserting the LuxI and LuxR genes into the vacant *htrA* locus in the bacterial genome, using the attenuated Salmonella (AIS) with *htrA* gene deletion as the backbone. Following intravenous injection, AISI‐H engineered bacteria accumulated and proliferated in tumors, secreted large quantities of AHL signaling molecules, and initiated the expression of the *htrA* gene. In normal organs, however, the density of AISI‐H engineered bacteria was low, failing to accumulate sufficient AHL signaling molecules and thus preventing *htrA* gene expression. This approach enables bacterial attenuation in normal organs while promoting toxic protein expression in tumors, thus achieving more precise and controllable bacterial tumor therapy [12].

Gao et al. utilized QSIs salvianolic acid A (SAA) and ractopamine (RH) to inhibit the AI‐2‐mediated QS process in *Streptococcus suis* by binding to the LuxS protein. This inhibition reduced EPS synthesis and the expression of bacterial adhesion‐related genes, thereby disrupting bacterial aggregation and biofilm structure [29]. Inhibiting AI‐2 QS can selectively reduce bacterial invasiveness and immune escape ability while retaining part of the bacteria's immunogenicity; this not only stimulates immune responses but also avoids risks associated with strong virulence.

These studies on the QS system have deepened understanding of bacterial colony behavior and virulence regulation mechanisms, and the ingenious application of QSIs has also opened new avenues for developing novel bacterial attenuation strategies (Table 1).

**TABLE 1:** Comparison of Attenuation Strategies for Engineered Bacteria.

Category	Specific Method	Operation	Advantage	Limitation	Representative example	References
Traditional Attenuation Strategy	Serial Passage	Forced adaptation under laboratory conditions leading to the progressive loss of virulence factors.	Simple operation and low cost.	Time‐consuming (often spanning years); unclear molecular basis; risk of virulence reversion; potential impairment of tumor‐targeting capacity.	BCG (*Bacillus Calmette‐Guérin*, an attenuated strain of *Mycobacterium bovis* used for bladder cancer).	[13]
	Chemical or Physical Mutagenesis	Random induction of genomic mutations to disrupt virulence genes.	Applicable for screening protective strains.	Highly stochastic with unpredictable outcomes; prone to damaging tumor‐targeting functions; complex safety assessment.	ATOM Sal‐L6 (γ‐ray irradiated Salmonella typhimurium).	[16]
Engineered Attenuation Strategy	Targeted Virulence Gene Knockout	Utilization of gene‐editing technologies (e.g., CRISPR‐Cas9) for the precise deletion of pathogenicity‐related genes.	High specificity; low risk of reversion; preservation of essential therapeutic functions.	Potential residual risks in immunocompromised hosts.	VNP20009: Deletion of *msbB* (to reduce endotoxicity) and *purI* (purine auxotrophy); ΔhtrA‐VNP: Deletion of *htrA* to reduce exopolysaccharides and increase macrophage sensitivity; *Acinetobacter baumannii*: Deletion of the *wza* gene to block capsular polysaccharide synthesis and thin the cell wall;	[12, 19, 28]
	Metabolic Pathway Modification	Deletion of essential nutrient biosynthesis genes, restricting bacterial survival to environments with specific supplements or the tumor site.	Tumor‐specific proliferation; significantly enhanced safety profile.	specific proliferation; significantly enhanced safety profile.Nutrient fluctuations in the tumor microenvironment may reduce colonization efficiency.	VNP20009: Deletion of *purI* impairs purine synthesis; Listeria: Deletion of *prfA*, rendering virulence gene activation dependent on glutamine and glutathione in the tumor microenvironment.	[21, 22, 23, 28]
	Quorum Sensing Regulation	Leveraging bacterial quorum‐sensing systems (e.g., LuxI/LuxR) to ensure virulence or therapeutic genes are expressed only when bacterial density reaches a specific threshold (i.e., after colonizing the tumor), while remaining silenced in blood or normal tissues (low density).	Enables dynamic attenuation; enhances safety.	Complex genetic circuit design; potential for off‐target effects; difficulty in threshold fine‐tuning.	AISI‐H: Insertion of the LuxI/LuxR system into Salmonella.	[12]

## 3 Challenges and Strategies for Efficacy Stability

Ensuring the stability of efficacy poses a significant challenge in the clinical implementation of attenuated bacterial therapy. Tumor heterogeneity and the dynamic nature of microenvironments often give rise to issues such as variations in bacterial colonization efficiency, uneven treatment responses, and challenges in sustaining efficacy [30, 31]. Variations in tissue structure, metabolism, and gene expression among patients with the same type of tumor are substantial. Disparities in blood vessel distribution, oxygen availability, and extracellular matrix composition at the tumor site result in several‐fold differences in bacterial colonization efficiency, leading to a phenomenon known as ‘local efficacy, overall inefficacy.

Beyond intratumoral heterogeneity, the deletion of metabolic genes or virulence factors during attenuation and genetic engineering—while significantly enhancing safety—inevitably compromises bacterial fitness and colonization capacity within the complex tumor microenvironment, thereby limiting the stability of therapeutic efficacy. Regarding Metabolic Auxotrophy, oncolytic bacteria are typically engineered as auxotrophs; for instance, the deletion of the *purI* gene in VNP20009 renders its proliferation highly dependent on exogenous adenine. Although this restricts growth primarily to necrotic regions, it also dictates that in peripheral tumor areas or nutrient‐deprived microenvironments, the colonization rate is substantially lower than that of wild‐type strains capable of de novo nutrient synthesis. Furthermore, the Loss of Immune Evasion remains a critical hurdle. Bacterial virulence factors often function as inherent defense mechanisms, and the common practice of truncating LPS (e.g., via *msbB* deletion) to mitigate systemic toxicity also strips the bacteria of protective barriers against the host complement system and phagocytes, significantly increasing the probability of “neutralization” within the systemic circulation before reaching the tumor.

In terms of Decreased Motility and Penetration, the tumor interior is characterized by exceptionally high interstitial fluid pressure (IFP), and the ATP‐intensive nature of bacterial chemotaxis is often compromised by the disruption of global energy homeostasis during attenuation. Consequently, attenuated strains may exhibit diminished flagellar motility compared to wild‐type counterparts, hindering their ability to navigate against pressure gradients into deep tumor tissues and resulting in insufficient colonization density. Finally, the Sensitivity to tumor microenvironment Stress further limits therapeutic potential. The tumor interior is an extreme environment characterized by acidosis and high levels of ROS. While wild‐type strains possess a sophisticated repertoire of stress‐response genes, interference with regulatory elements such as specific transporters during the attenuation process weakens bacterial resistance to oxidative damage. This results in a phenotype where the bacteria may successfully infiltrate the tumor but fail to maintain long‐term viability, effectively “entering but not persisting” within the target tissue.

Consequently, targeted interventions aimed at modulating bacterial colonization, survival, and functional expression are imperative to enhance the stability of efficacy and the clinical viability of attenuated bacterial therapy.

### 3.1 Intelligent Responsive Engineering Bacteria

Tradititional therapies struggle to adapt to the dynamic changes in the tumor microenvironment due to their limited regulatory capacity. By developing engineered bacteria with intelligent responsive capabilities, precise control over bacterial colonization and biological functions can be achieved, leading to enhanced and consistent therapeutic outcomes.

#### 3.1.1 Response to External Physical Signals

Qiao et al. developed near‐infrared light‐responsive oncolytic bacteria by embedding an optogenetic element (PadC protein) into the Salmonella genome to construct the NETMAP system. Under near‐infrared light (710 nm) irradiation, the bacteria can produce immune checkpoint inhibitors (e.g., PD‐L1 antibodies) or cytotoxic proteins (e.g., ClyA) on demand, enabling differential treatment of high/low immunogenic tumors [32].

**FIGURE 5:**
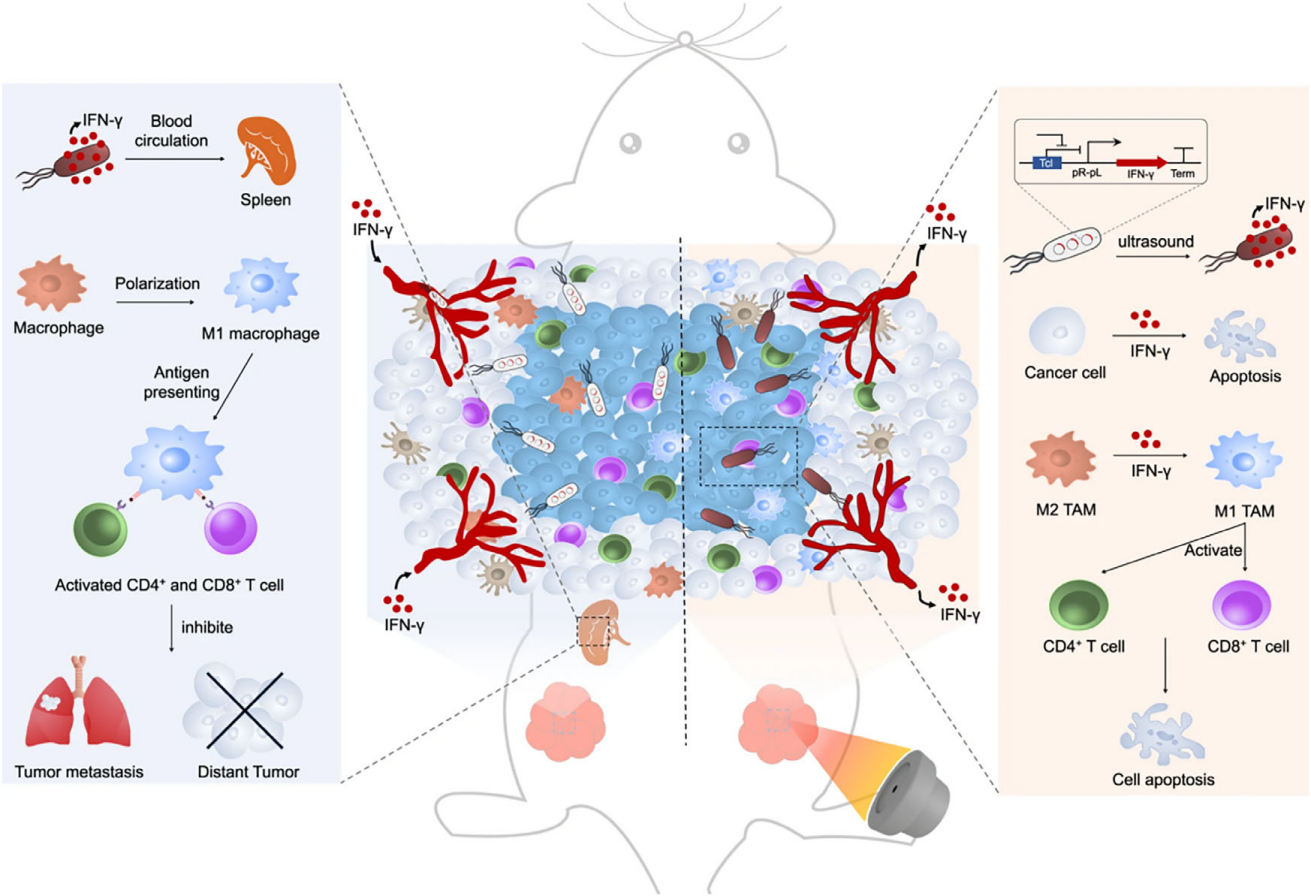
Schematic diagram of URB in controlling IFN‐γ expression by focused ultrasound and their mechanisms for cancer immunotherapy [33]. Upon systemic administration of URB that contains IFN‐γ gene inserted in the temperature‐sensitive genetic circuit, these genetically engineered bacteria would deliver into the tumors due to their tumor‐targeting capability. Then, the tumor was irradiated by focused ultrasound to heat these intratumoral bacteria to 42°C–45°C for inducing the expression of IFN‐γ gene. The production and secretion of IFN‐γ not only can promote the apoptosis of cancer cells, but also induce the macrophage polarization from M2 to M1 phenotype and the activation of CD4+ and CD8+ T cells (Right panel).In addition, high levels of IFN‐γ can also activate macrophages and T cells in the spleen to inhibit lung metastasis and distant tumor growth through immune memory responses (Left panel). Reproduced with permission [33]. Copyright 2022, Springer Nature.

Chen et al. constructed an ultrasound‐responsive bacterium (URB) by introducing a plasmid containing a temperature‐sensitive pR‐pL promoter into E. coli MG1655. Under the action of the heat‐labile cI857 repressor protein, IFN‐γ transcription is inhibited at low temperatures. Ultrasound converts mechanical energy into heat energy, and expression is initiated when the local tumor temperature rises to 42°C–45°C, thereby enabling spatiotemporal regulation of therapeutic factors (Figure 5) [33].

The magnetic control lysis system modifies magnetic nanoparticles (Fe_3_O_4_) on the bacterial surface. Under an alternating magnetic field, the engineered bacteria can locally heat up, trigger the expression of lytic proteins controlled by a thermosensitive promoter, release anti‐CD47 nano‐antibodies, enable spatiotemporal control of drug release, and activate the type I interferon pathway in antigen‐presenting cells via bacterial lysates to enhance adaptive immunity [34].

Li et al. designed and prepared a bimetallic nanoenzyme‐coated bacterial vector (Au‐Pt@VNP20009, APV). By utilizing a strong tumor inflammatory response induced by low‐dose X‐rays, they enhanced the ability of CD11b^+^ immune cells to assist APV in targeted tumor delivery, thereby effectively alleviating tumor hypoxia and immunosuppression, and inhibiting tumor growth and metastasis [35].

#### 3.1.2 Response to External Chemical Signals

Regulation based on exogenous chemical signals confers a high degree of manual controllability to bacterial therapy. Utilizing small‐molecule inducers (e.g., tetracycline, IPTG) as regulatory switches allows clinicians to modulate the timing of therapeutic initiation and dosage according to the patient's status, facilitating precise and efficient management of the treatment process.

Tetracycline and its derivatives induce conformational changes in the TetR repressor protein through high‐affinity binding, causing it to dissociate from the promoter sequence. This relieves repression and activates downstream gene expression. Jiang et al. successfully engineered Salmonella typhimurium to express therapeutic payloads and imaging reporter genes under the control of a tetracycline‐inducible promoter (Ptet). In this system, gene expression is stringently silenced by TetR under normal physiological conditions and is activated only following the systemic administration of doxycycline (Dox), thereby achieving precise temporal control and significantly mitigating systemic off‐target toxicity [36].

IPTG, a non‐metabolizable lactose analogue, specifically binds to and inactivates the LacI repressor, thereby efficiently initiating transcription of genes downstream of the lac operon. Harimoto et al. developed a programmable capsular polysaccharide (CAP) expression system in *Escherichia coli* Nissle 1917. In this system, the expression of surface CAP is driven by an IPTG‐inducible promoter. Systemic administration of IPTG triggers the formation of a protective CAP layer, enabling the bacteria to evade immune clearance and safely reach the tumor. The withdrawal of IPTG results in the loss of the CAP layer, leading to rapid bacterial clearance and significantly enhancing therapeutic safety [37].

Vanillate, a plant‐derived small molecule, has been developed as an excellent orthogonal regulatory tool due to its lack of interference with endogenous bacterial metabolism and absence of cytotoxicity. Vanillate relieves the inhibition of the promoter by the repressor protein VanR. In constructing the “Marionette” E. coli strain, Meyer et al. employed protein engineering to precisely modify the amino acid sequence of VanR, substantially increasing its sensing sensitivity. Research indicates that the vanillate‐inducible system exhibits exceptionally high expression efficiency and can be used alongside eleven other small‐molecule inducers (such as IPTG and salicylate) to trigger distinct genetic functions without crosstalk. This highly specific switching mechanism provides the technical foundation for the simultaneous and independent control of multiple therapeutic agents within the same bacterium [38].

AHL is a key signaling molecule in bacterial quorum sensing; its concentration accumulates as population density increases, and upon reaching a critical threshold, it binds to the LuxR protein to initiate specific gene expression. Din et al. constructed a synchronized lysis circuit (SLC) in Salmonella based on the LuxI/LuxR system. In this circuit, the accumulation of AHL acts as a chemical signal that, once the critical population threshold is reached, triggers the expression of a bacteriophage lysis gene (phiX174 E). This leads to periodic bacterial lysis and the release of therapeutic payloads—a mechanism that maximizes efficacy while autonomously limiting total bacterial burden to prevent toxicity [39].

In summary, bacterial expression regulatory systems are evolving from classical exogenous controls (e.g., tetracycline, IPTG) toward multiplexed orthogonal regulation (e.g., vanillate) and autonomous intelligent feedback (e.g., AHL‐mediated quorum sensing). This progression is driving the development of bacterial therapies into sophisticated, “intelligent” living medicines.

#### 3.1.3 Response to Tumor Microenvironment Signals

Bacterial environmental response mechanisms enable sensing and adaptation to external signals such as pH, temperature, oxidative stress, and nutrient availability. By incorporating regulatory elements that detect these signals into engineered strains, precise control of therapeutic gene expression (e.g., toxins or immunostimulators) can be achieved in specific microenvironments, such as the acidic or high‐temperature conditions found in tumors.

Lactic acid concentrations in the tumor microenvironment (>5 mM) are significantly higher than those in normal tissues (≈1.5 mM), a feature that can be harnessed to enable targeted bacterial colonization. Zou et al. constructed an engineered strain, EcNlEACS, based on *Escherichia coli* Nissle 1917. A lactate sensor was first developed using the LldR gene from Corynebacterium glutamicum to ensure that EcNlEACS survives exclusively under high lactate conditions (>5 mM) specific to the tumor microenvironment and avoids colonizing normal tissues [40].

The acidic pH of the tumor microenvironment also provides a precise regulatory signal. Do et al. investigated the interaction between the quorum sensing peptide (SIP) secreted by *Streptococcus pyogenes* (GAS) and its intracellular receptor RopB in regulating the expression of the virulence factor SpeB. SIP is secreted and enters the cell at high bacterial density. The pH‐sensitive histidine (H144) in RopB can sense environmental acidity. In a neutral environment, H144 is not protonated, and its interaction with surrounding amino acids is unstable, leading to weak binding of RopB to SIP. This inhibits SpeB transcription and zymogen maturation, thereby reducing GAS virulence [41]. Additionally, bacterial self‐destruction systems can be constructed based on environmental response mechanisms, such as the pH‐sensitive kill switch (acidTRP) developed by Stirling et al. Under acidic conditions (pH 5), acidTRP activates the expression of the Doc gene; the Doc protein inhibits bacterial protein translation via the phosphorylation of EF‐Tu, ultimately leading to bacterial death [42].

Hypoxia and elevated ROS are hallmark features of the tumor core. Qiao et al. induced ferroptosis in pancreatic ductal adenocarcinoma using engineered E. coli (ZZY@RM) expressing cystathionine lyase (CGL) under a hypoxia‐responsive promoter, depleting cysteine specifically in hypoxic regions and avoiding off‐target effects [43]. Xie et al. employed the hypoxia‐responsive *fdhF* promoter in attenuated E. coli MG1655 to drive CD47 nanobody expression, blocking the CD47‐SIRPα axis only in hypoxic tumors. Combined with GSH‐responsive liposomes releasing M‐CSF, this strategy recruits and activates macrophages to overcome immune suppression [44].

The intrinsic thermal signatures resulting from the robust metabolism of tumor cells provide another natural stimulus for precision regulation. Twittenhoff et al. controlled the translation of the cytotoxic necrosis factor gene cnfY via temperature‐sensitive RNA structures in the 5'‐untranslated region (5'‐UTR). These structures fold into stem‐loop configurations at low temperatures, masking ribosome binding sites (RBS) and thus preventing the translation of toxin genes. By genetically engineering RNA thermometers using this temperature‐sensitive 5'‐UTR variant, the stem‐loop structure is maintained at normal body temperature, shielding the RBS to inhibit virulence gene expression and prevent damage to normal tissues. Conversely, at the specific temperatures of tumor regions, the structure unwinds to initiate translation [45].

Notably, the interaction between bacteria and the microenvironment is not unidirectional. Beyond passively sensing physicochemical signals, colonizing bacteria can actively reshape the immunological landscape of the TME. For instance, Kaimala et al. demonstrated that Salmonella can polarize myeloid‐derived suppressor cells (MDSCs) toward a pro‐inflammatory phenotype via the MyD88–TLR pathway, effectively overcoming immune tolerance and inducing tumor regression. This study provides significant insights, suggesting that bacterial therapeutic strategies targeting myeloid immunosuppressive cells serve as a potent means of activating antitumor immunity [46, 47].

Intelligently responsive engineered bacteria overcome the limitations of traditional tumor therapies via signal‐driven regulation, enabling precise bacterial control. They can also effectively regulate the tumor microenvironment, reshape its immunosuppressive state, and further activate the body's immune response.

### 3.2 Combination Therapy Strategy

Attenuated bacterial monotherapy exhibits limited efficacy in clinical settings, necessitating the exploration of multimodal synergistic therapies. When combined with other treatments, attenuated bacteriotherapy can integrate the advantages of diverse therapies and significantly enhance antitumor efficacy.

#### 3.2.1 Bacteria Combined with Chemotherapy and Non‐Antitumor Drugs

Engineered bacterial strains not only reverse chemotherapy resistance by activating quiescent tumor cells but also augment the antitumor efficacy of drugs by modulating the tumor microenvironment. Furthermore, while conventional chemotherapeutic agents struggle to penetrate the hypoxic core of tumors and often induce systemic toxicity, oncolytic bacteria specifically colonize these regions. Consequently, combining bacteria with chemotherapy enables targeted synergistic enhancement.

First, bacteria can sensitize quiescent tumor cells that are typically resistant to chemotherapy. Yano et al. demonstrated that the tumor‐targeting Salmonella A1‐R strain can transition tumor cells from the quiescent phase (G0/G1) back into the active cell cycle (S/G2/M). By forcing these dormant cells into the proliferative phase, the bacteria render them highly sensitive to subsequent cytotoxic chemotherapy (e.g., cisplatin or paclitaxel), thereby significantly enhancing the inhibitory effect of the combination therapy [48]. Tumor‐targeting Salmonella typhimurium A1‐R, in combination with recombinant methioninase, demonstrates significant tumor inhibition in a doxorubicin‐resistant DDLPS PDOX model. This finding suggests that bacteria‐based metabolic intervention strategies may offer a novel therapeutic approach for rescuing cases of chemotherapy failure [49].

Second, the alterations in the tumor microenvironment triggered by bacterial accumulation create conditions that allow non‐antitumor drugs to exert potent anticancer activity. Research has found that intravenous bacterial injection induces increased secretion of systemic TNF‐α, leading to the disruption of tumor vasculature and the induction of tumor‐specific hemorrhage. Subsequently, tumor‐associated macrophages (TAMs) phagocytose erythrocytes that have infiltrated the tumor. Although this process initially triggers pro‐inflammatory polarization of TAMs, this phenotype is often unsustainable due to the rapid degradation of erythrocytes. Xu et al. discovered that the co‐administration of the antimalarial drug artesunate effectively maintains the pro‐inflammatory phenotype of TAMs. The synergistic mechanism lies in the ability of artesunate to form complexes with heme released from lysed erythrocytes; these complexes continuously catalyze the production of ROS, thereby sustaining the pro‐inflammatory state of TAMs and exerting antitumor effects. Notably, low‐dose artesunate lacks significant antitumor activity as a monotherapy, but when combined with bacteria‐induced hemorrhage, it markedly enhances overall therapeutic outcomes [50].

This biological effect has also been extended to enable the anti‐osteoporosis drug zoledronic acid to exert antitumor functions. Under conditions of bacteria‐induced hemorrhage, Xu et al. observed that tumor cells also exhibit erythrophagocytic behavior, resulting in the intracellular release of high concentrations of ferrous ions (Fe^2+^). Zoledronic acid specifically captures these enriched intracellular Fe^2+^ions through coordination reactions, leading to the in situ self‐assembly of iron‐zoledronic acid nanoparticles. This process triggers an explosive generation of ROS, directly inducing tumor cell apoptosis [51].

#### 3.2.2 Bacteria Combined with Radiotherapy

The synergy between bacteria and radiotherapy aims to leverage the immunostimulatory properties and inherent motility of bacteria to enhance the presentation of tumor antigens following irradiation. Although radiotherapy can release a vast reservoir of tumor antigens by inducing immunogenic cell death (ICD), effective antigen presentation is often hindered by the presence of immunosuppressive factors within the tumor microenvironment, which impair dendritic cell (DC) function. The introduction of bacteria serves not only as a potent adjuvant to activate innate immunity but also as an active transport mechanism, ensuring that radiotherapy‐released antigens are effectively recognized by the immune system to augment antitumor outcomes.

Studies have demonstrated that following tumor radiotherapy, cation‐modified attenuated Salmonella can capture the antigens released by irradiation and, utilizing bacterial motility, transport these antigens to the tumor periphery. This process facilitates antigen delivery to functional DCs surrounding the tumor, thereby enhancing their antigen‐presenting capacity and eliciting a systemic antitumor immune response [52]. Furthermore, Lim et al. employed an attenuated *Listeria monocytogenes* vaccine (ΔactA/ΔinlB strain) expressing a model tumor‐associated antigen (OVA) in combination with a high‐dose single‐fraction (15 Gy) radiotherapy to treat a B16 melanoma model. The study revealed that radiotherapy induces IFN‐γ production and upregulates the expression of MHC class I molecules and tumor‐associated antigens (TAAs) on tumor cells. The bacterial vaccine significantly increased the intratumoral infiltration of antigen‐specific CD8+ T cells, total T cells, and NK cells. These elevated T cells effectively recognized the radiotherapy‐upregulated tumor antigens, thereby optimizing antitumor activity. The complementary activation of innate and adaptive immunity resulted in a synergistic tumor control effect that was markedly superior to monotherapy and successfully induced long‐term immune memory in a subset of mice [53].

#### 3.2.3 Bacteria Combined with Immunotherapy

Bacteria can be combined with various immunotherapies, such as immune checkpoint inhibitors (ICIs) and CAR‐T cell therapy. ICIs can mitigate the adverse effects resulting from the bacteria‐induced upregulation of PD‐L1 expression. Conversely, bacteria can significantly increase the intratumoral concentration of ICIs or cytokines through site‐specific delivery, thereby reducing systemic adverse reactions. Furthermore, bacteria can enhance the antitumor efficacy of CAR‐T cells by synthesizing universal synthetic antigens.

Research indicates that attenuated Salmonella can activate inflammatory signaling pathways within the tumor microenvironment. This robust inflammatory response induces a compensatory upregulation of PD‐L1 on tumor cells, which inhibits T cell activity. PD‐L1 inhibitors can resolve this bacteria‐induced suppression, thereby augmenting the efficacy of bacterial cancer therapy [54]. Additionally, Gurbatri et al. engineered *Escherichia coli* Nissle 1917 harboring a SLC to autonomously lyse upon reaching a critical density, ensuring the continuous intratumoral release of PD‐L1 and CTLA‐4 nanobodies. By secreting these nanobodies directly within the tumor, this platform significantly elevates local checkpoint inhibitor concentrations while bypassing the autoimmune side effects associated with systemic administration, ultimately inducing a more potent antitumor response [55]. Similarly, utilizing bacteria such as Salmonella as delivery vehicles for cytokines (e.g., IL‐2) markedly reduces systemic toxicity [56].

To address the challenge of antigen heterogeneity in solid tumors, bacterial vectors offer unique advantages. To overcome immune escape caused by non‐uniform antigen expression, Danino et al. developed a probiotic‐guided CAR‐T platform. They utilized E. coli Nissle 1917 to specifically colonize the tumor core and continuously secrete synthetic antigens tagged with a collagen‐binding domain. These antigens anchor firmly to the tumor stroma, allowing intravenously injected CAR‐T cells—engineered to recognize these synthetic antigens—to exert their antitumor effects. This approach effectively bypasses the issue of endogenous antigen heterogeneity [57]. Furthermore, studies have found that depleting neutrophils via IFN‐γ or anti‐Granulocyte differentiation antigen‐1 (Gr‐1) antibodies can dismantle the physical barriers formed by neutrophils that impede bacterial dispersion, thereby enhancing the efficacy of bacterial antitumor therapy [58, 59].

#### 3.2.4 Multi‐Strain Bacterial Cocktails

The division of labor in synthetic flora refers to the design of functional differentiation and cooperation among different microbial strains, enabling them to collectively accomplish complex tasks. The core involves constructing a multi‐strain collaborative system via engineering approaches, utilizing metabolic functional complementarity among bacterial populations, signal molecule regulation, and spatial distribution design to enhance the efficacy of single strains or achieve complex functions that cannot be accomplished by a single strain.

Zhou et al. constructed a synthetic bacterial community (SynCon) consisting of multiple engineered E. coli strains. These strains harbor distinct sensing modules that detect signals in the tumor microenvironment, including lactic acid, pH, and hypoxia, and are activated upon the occurrence of corresponding signal changes in the tumor microenvironment. The coordinated operation of sensing modules across multiple strains enables SynCon to sense the characteristics of the tumor microenvironment more comprehensively and accurately, thereby enhancing specific tumor recognition. Meanwhile, distinct strains within SynCon harbor unique therapeutic payloads that can synergistically inhibit tumor growth through various mechanisms (e.g., inducing apoptosis and recruiting immune cells), thereby enhancing therapeutic efficacy [60].

Montalban‐Arques et al. orally administered CC4, a mixture of four Clostridium bacteria, to tumor‐bearing mice. Owing to the infiltration and activation of CD8^+^ T cells within tumors, the CC4 mixture exhibited a more potent antitumor effect in the MC‐38 and B16 melanoma models compared to other control groups, indicating that these strains exert a synergistic effect in inhibiting tumor growth [61].

Bacterial conjugation is primarily mediated by the Type IV Secretion System (T4SS), which facilitates the potential dissemination of genetic material between strains. To mitigate the risks associated with the emergence of novel strains through conjugation, several countermeasures have been developed. A pivotal approach involves the physical obstruction of conjugation channels using specific small‐molecule inhibitors. TraE, a core inner‐membrane protein of the T4SS, serves as a central node in DNA transfer by binding DNA with high affinity and forming the translocation channel through oligomerization and interactions with TraD and VirB4. Research has demonstrated that BAR‐072, an unsaturated fatty acid derivative, can specifically inhibit the DNA‐binding activity of TraE, thereby directly blocking the loading and transport of DNA [62]. By disrupting the interaction between essential transport proteins and substrate DNA, this strategy effectively severs the molecular pathway of plasmid conjugation at its mechanical root.

Furthermore, the implementation of obligatory metabolic interlocking systems serves as an additional safeguard to mitigate conjugation‐associated risks. By engineering unique and complementary essential auxotrophies within the microbial community—for instance, an *argC* deletion strain (ΔargC) deficient in arginine biosynthesis and a *metA* deletion strain (ΔmetA) deficient in methionine biosynthesis—[63] each strain becomes strictly dependent on metabolites provided through reciprocal cross‐feeding. While this strategy does not directly prevent bacterial conjugation, it imposes a stringent ecological constraint: any individual cell that escapes the consortia will fail to survive or proliferate due to the absence of essential metabolic complements. Consequently, this dependency effectively limits the dissemination of any novel transconjugants by ensuring their survival is contingent upon a specific microenvironment, thereby reducing biosafety risks.

Rational utilization of the division of labor in synthetic flora, based on its unique multi‐species synergy mechanism, can reduce the metabolic burden on individual bacteria and enhance metabolic efficiency. Different bacteria undergo distinct attenuation treatments.

### 3.3 Interactions Between Therapeutic Bacteria and Host Resident Microbiota

While oncolytic bacteria exert therapeutic effects through diverse mechanisms, their efficacy is significantly influenced by resident intratumoral and gut microbiota. These indigenous microbes interfere with therapeutic outcomes by competing for colonization niches and nutrients, facilitating horizontal gene transfer, and modulating the systemic immune landscape.

The necrotic and hypoxic regions of tumors are the preferred colonization sites for oncolytic bacteria. However, resident intratumoral bacteria (e.g., Fusobacterium nucleatum and members of the Enterobacteriaceae family) often establish mature biofilms within these core regions. Such physical barriers not only occupy potential colonization niches but also restrict the deep diffusion of oncolytic bacteria throughout the tumor tissue. Furthermore, indigenous tumor‐resident microbes have evolved to become highly adapted to the tumor microenvironment, enabling them to sequester limited trace elements (e.g., iron, zinc) and carbon sources more efficiently than therapeutic strains, thereby creating intense nutritional competition. These resident microbes may also transfer genetic material to oncolytic bacteria through conjugation, potentially compromising their genetic stability. Additionally, the composition of the gut microbiota dictates the host's immune status. By continuously modulating systemic immunity via TLR (Toll‐like receptor) signaling pathways, the gut microbiota can reduce the survival of oncolytic bacteria in vivo and impair their subsequent colonization within the tumor.

To address these challenges, strategies must be developed to mitigate the interference from resident intratumoral and gut microbiota. Oncolytic bacteria can be engineered to secrete dispersins or matrix‐degrading enzymes to disrupt indigenous biofilms, thereby enhancing bacterial penetration and tumor colonization. Moreover, the metabolic repertoire of therapeutic strains can be expanded to utilize non‐competitive carbon sources. Through synthetic biology, bacteria can be programmed to metabolize substrates that are abundant in the tumor but inaccessible to resident microbes, such as specific D‐amino acids or tumor‐unique glycolytic intermediates. To mitigate the risk of genetic conjugation, T4SS inhibitors can be employed to block conjugation channels, or CRISPR‐Cas‐based anti‐conjugation systems can be integrated into oncolytic strains. Regarding the systemic immune clearance driven by gut microbiota, therapeutic bacteria can be engineered with modified CAP to evade recognition by phagocytes or cross‐reactive antibodies induced by commensal gut microbes [37].

In summary, the native gut and tumor‐resident microbiome are directly linked to the colonization stability and clinical efficacy of bacterial cancer therapies. Elucidating and managing the complex interactions between engineered therapeutic bacteria and the indigenous microbiota is a critical prerequisite for successful clinical translation.

### 3.4 Genetic and Functional Stability of Attenuated Phenotypes

The distinct characteristics of the tumor microenvironment, such as hypoxia, acidosis (low pH), and elevated levels of reactive oxygen species, may compromise the stability of attenuated bacteria. Consequently, it is imperative to employ multifaceted strategies to maintain bacterial stability during intratumoral proliferation. First, the complete deletion of essential genes or operons from the bacterial chromosome is required. Once large genomic fragments are physically excised, it becomes virtually impossible for the bacteria to reacquire the lost base pairs through random mutations within a short timeframe, thereby ensuring high genetic stability. A representative example is the VNP20009 strain, which features deletions of the *msbB* and *purI* genes to reduce endotoxicity and establish adenine dependence, respectively. Because these large‐scale deletions are permanent, the strain maintains its low‐toxicity phenotype even during extensive expansion within the tumor. Second, the engineering of auxotrophic strains ensures that bacterial growth is contingent upon substrates—such as specific amino acids, adenine, or uracil—that are scarce in healthy tissues but abundant in the necrotic regions of tumors. VNP20009 specifically leverages the high adenine concentrations within the tumor to proliferate, whereas its survival is severely restricted in normal tissues. This metabolic dependency aligns bacterial growth with the available nutritional resources of the tumor, thereby enhancing the functional and phenotypic stability of the therapeutic agent [17].

Furthermore, synthetic biology approaches can be employed to couple the deletion of virulence genes with the retention of essential bacterial genes. For instance, Sun et al. integrated an essential metabolic gene, such as one involved in tryptophan biosynthesis, into the locus where the Salmonella virulence gene sseJ had been excised. This configuration enables the bacterium to synthesize the essential metabolite required for survival while maintaining its attenuated state. This design leverages the physical, mutually exclusive relationship between the virulence gene and the essential gene: any homologous recombination event leading to the reversion of the wild‐type sseJ virulence gene would inevitably result in the loss of the essential metabolic gene occupying that site. Consequently, any mutation restoring virulence would compromise core physiological functions and lead to bacterial death, thereby ensuring the robust long‐term stability of the attenuated strain [64].

To circumvent the release of genetic material when engineered bacteria encounter survival pressures, a dual reduction strategy targeting both chromosomes and plasmids can be adopted. First, at the host genome level, homologous recombination techniques are utilized to excise non‐essential regions, such as transposons, insertion sequences (IS) that trigger genomic instability, and redundant secondary metabolic gene clusters [65]. For instance, the *Escherichia coli* strain DGF‐298 underwent a 1,670 kb (35.9%) genomic reduction, which successfully minimized metabolic competition and genetic instability while enhancing the expression efficiency of core pathways. Furthermore, de novo genome design and chemical synthesis enable the construction of minimal genomes, as demonstrated by Mycoplasma JCVI‐syn3.0 (531 kb), which proves the feasibility of maintaining viability with a streamlined genetic blueprint. To alleviate the metabolic burden imposed by plasmid redundancy, the DdmDE system can be employed to refine plasmid content [66]. In this system, the nuclease DdmE is guided by short guide DNAs (gDNAs) designed to target unique sequences within redundant plasmids, such as helper or non‐essential cloning vectors. Upon DNA binding, DdmD transitions from an inactive dimer to an active monomer, leveraging its helicase and nuclease activities for the continuous degradation of the targeted plasmids. This pre‐programmed recognition mechanism ensures the precise elimination of redundant vectors, allowing the bacteria to maintain only the minimal therapeutic payload required for efficacy.

It is noteworthy that the biochemical persistence of genetic material released following bacterial lysis is extremely limited within the host environment. Human blood, interstitial fluids, and mucus are abundant in various nucleases, such as DNase I. Once bacterial DNA enters the circulatory system, it typically exhibits an exceptionally short half‐life and is rapidly degraded into innocuous nucleotide monomers [67]. This efficient clearance mechanism ensures that the probability of exogenous naked DNA integrating into the host genome remains negligible under physiological conditions [68]. Furthermore, the release of genomic components from bacteria is not entirely a deleterious factor in the context of oncotherapy. On the contrary, released DNA can function as a potent natural immunoadjuvant. This exogenous DNA is recognized by host pattern recognition receptors (PRRs), which subsequently activate innate immune responses in situ via the TLR9 signaling pathway and the intracellular cGAS‐STING sensing pathway. This activation assists in remodeling the immunosuppressive tumor microenvironment, thereby augmenting anti‐tumor efficacy [69, 70]. Although DNA released from bacterial lysis is typically cleared rapidly in vivo, proactive genetic intervention strategies remain necessary to enhance biosafety and engineering controllability.

In summary, by employing large‐scale genomic deletions, auxotrophy, and virulence–essential gene coupling, combined with the dual streamlining of chromosomes and plasmids, the risks of phenotypic reversion and genetic material release can be mitigated within the complex tumor microenvironment, thereby enhancing the genetic and functional stability of attenuated bacteria.

### 3.5 Predictive Biomarkers for Patient Stratification in Bacterial Cancer Therapy

The primary barrier to the clinical translation of bacterial therapy lies in the profound inter‐patient heterogeneity of tumor physiology. Unlike standard radiotherapy or chemotherapy, bacterial colonization is an active biological process strictly constrained by specific environmental and nutritional conditions within the tumor microenvironment. These include hypoxic cores conducive to anaerobic survival and specific nutritional substrates, such as purines and amino acids, required by auxotrophic strains. Consequently, variations in baseline tumor microenvironment characteristics lead to inconsistencies in bacterial load and therapeutic response across patients. To overcome this challenge, identifying predictive biomarkers for patient stratification is essential.

First, the metabolic profile of a tumor is a direct determinant of successful bacterial colonization. Since auxotrophic bacteria depend on tumor‐derived nutrients, specific metabolic signatures dictate colonization efficiency. For instance, a high lactate/pyruvate ratio—a hallmark of the Warburg effect [71] and severe hypoxia—[72] indicates both a low‐oxygen environment suitable for anaerobes and the availability of specific carbon sources required for bacterial growth. Thus, this ratio serves as a robust predictor for the successful colonization of obligate or facultative anaerobes, such as Salmonella [73].

Second, the status of the tumor immune microenvironment is critical to the success of bacterial therapy. High intratumoral expression of IDO (indoleamine 2,3‐dioxygenase) typically indicates deep immunosuppression dominated by MDSCs, serving as a predictive indicator for assessing bacterial colonization capacity [74]. IDO catalyzes the degradation of tryptophan into kynurenine; this process inhibits T cell proliferation and effector functions through local tryptophan depletion while simultaneously activating the AhR signaling pathway via kynurenine to maintain an MDSC‐dominated immunosuppressive tumor microenvironment. Targeted antagonism of IDO can reactivate suppressed T cells and attenuate MDSC‐mediated immunosuppression, thereby effectively triggering the antitumor immune responses initiated by bacteria [75].

Finally, the physical characteristics of the tumor extracellular matrix (ECM) constitute a key barrier limiting deep bacterial penetration. Intratumoral bacterial distribution is not a matter of simple passive diffusion but depends on active flagella‐driven motility. Collagen density and matrix stiffness directly determine the efficiency of this movement. In highly fibrotic tumors, dense collagen fiber networks form a rigorous physical sieve that traps bacteria in perivascular peripheral regions, preventing them from migrating to the deep necrotic core favorable for proliferation [76]. Utilizing elastography to assess matrix stiffness can identify patients with physical barriers to colonization, suggesting potential insensitivity to bacterial therapy or the need for pre‐treatment with anti‐fibrotic agents to facilitate bacterial infiltration [77]. Consequently, tumor matrix stiffness serves as an effective metric for evaluating bacterial therapy potential.

Therefore, clinical trials should not regard bacterial therapy as a universal treatment modality. Instead, pre‐treatment screening based on metabolic imaging or biopsy profiling should be implemented. Correlating these biomarkers with tumor‐specific bacterial accumulation will assist clinicians in identifying specific patient populations most likely to benefit, thereby driving the transition of bacterial therapy toward a precision medicine paradigm (Table 2).

**TABLE 2:** Challenges and Strategies for Therapeutic Stability.

Category of Challenge	Primary Challenges	Corresponding Strategies
Dynamic Tumor Microenvironment Fluctuations	Hypoxia, acidic pH, high ROS, inflammation, and nutrient fluctuations interfere with bacterial metabolism, survival, and therapeutic cargo secretion, leading to significant inter‐individual variation in efficacy.	Environmental signal‐responsive systems (driven by pH, hypoxia, lactate, temperature, etc.); suicide switches; metabolic pathway optimization.
Inherent Limitations of Attenuation and Engineering	Deletion of key virulence genes impairs immune evasion mechanisms; genetic modifications may interfere with chemotactic receptors or flagellar motility, limiting bacterial infiltration into the tumor core; exogenous gene expression imposes a metabolic burden, compromising growth and adhesion capabilities.	Precise gene knockout coupled with essential gene linkage; multiple auxotrophy; synchronized lysis circuits (SLC) to prevent mutation propagation.
Tumor Heterogeneity and Patient Variability	Significant disparities in tissue architecture, metabolism, and gene expression among patients with the same tumor type; variations in vascularization, oxygen availability, and extracellular matrix (ECM) composition lead to substantial differences in colonization efficiency.	Multi‐signal integration circuits; identification of predictive biomarkers for patient stratification.
Indigenous Microbiota Interference	Tumor‐resident bacteria compete for colonization niches and nutrients or undergo genetic conjugation; the gut microbiota modulates systemic immunity, potentially suppressing the survival of engineered strains.	Expression of dispersins to disrupt resident bacterial biofilms; utilization of non‐competitive carbon sources; anti‐conjugation systems; regulated CAP expression.
Risk of Genetic Material Release	Selective pressure or bacterial lysis leads to the release of genetic material, posing risks of genomic integration or adverse immune responses; multiple plasmids or non‐essential genomic regions increase metabolic burden and instability.	Dual streamlining of chromosomes and plasmids (deletion of non‐essential regions/minimal genome); DdmDE system for the clearance of redundant plasmids.

## 4 Bacterial In Vivo Detection and Imaging Techniques

To some extent, the aforementioned schemes provide robust support for improving the stability of efficacy; however, the actual application effect of these strategies largely depends on the accurate understanding of bacterial colonization, distribution, activity, and metabolic status in vivo. Owing to the complexity of the in vivo environment, bacterial behavior in vivo is dynamically influenced by multiple factors, which may result in inadequate prediction of efficacy fluctuations.

In vivo bacterial detection and imaging technologies can clearly visualize the targeted colonization efficiency, survival status, and host‐bacterial interactions by enabling real‐time visual tracking, quantitative analysis, and dynamic monitoring of bacteria in vivo, thereby providing a direct basis for evaluating therapeutic efficacy. If issues such as abnormal bacterial distribution or activity attenuation in vivo are identified, the treatment regimen can be adjusted promptly to further ensure the stability of efficacy and provide key technical support for the smooth progression of clinical translation.

### 4.1 Optical Imaging

Optical imaging enables localization by capturing optical signals produced or carried by bacteria, and it offers advantages such as high sensitivity and spatiotemporal resolution. Based on the generation mechanism of bacterial luminescence signals, optical imaging can be divided into three technical branches: those relying on luciferase, fluorescent proteins, and exogenous fluorescent probes.

#### 4.1.1 Genetically Encoded Reporters

This approach involves transforming bacteria with genes encoding optically active proteins (e.g., luciferase, fluorescent proteins) via genetic engineering. The signal generation depends on the bacteria's own protein expression machinery.

Integrating luciferase‐encoding genes (e.g., Lux, Fluc [78]) into bacterial genomes enables autoluminescent imaging without external excitation light, suitable for deep tissue detection. Wu et al. constructed an attenuated Salmonella strain, VNP‐LuxCDABE, which expresses luciferase (LuxCDABE). They labeled macrophages with the near‐infrared fluorescent dye DiR and then loaded the VNP‐LuxCDABE strain into these macrophages to achieve dual labeling. The near‐infrared fluorescence signal of DiR reflects the targeted migration and aggregation of macrophages to tumors, thereby indicating the bacterial delivery route. In contrast, the bioluminescent signal of Lux directly indicates the distribution range of bacteria within tumors, and changes in its intensity can dynamically reflect bacterial proliferation after release from macrophages [79].

Bacteria can also be engineered to express fluorescent proteins or tags that light up upon excitation or ligand binding. Direct Panteli et al. genetically engineered attenuated Salmonella to express the fluorescent protein ZsGreen. When the bacteria colonize the tumor, they are induced to express ZsGreen, which is then released into the tumor microenvironment. Bacterial distribution and fluorescence signals are detected via fluorescence imaging technology [80]. Guo et al. constructed attenuated Salmonella typhimurium expressing the fluorescence activation protein dL5 (FAP dL5) and developed photodynamic therapy (PDT)‐enhanced oncolytic bacterial immunotherapy (OBI). This therapy can induce FAP dL5 expression in tumor tissues via L‐arabinose. Upon combination with exogenous malachite green (MG) or diiodinated MG (MG‐2I), it not only enables the detection of strong fluorescence signals and specific imaging in tumor tissues but also generates ROS to induce oxidative stress, thereby effectively killing tumor cells with excessive bacterial colonization [81].

#### 4.1.2 Exogenous Chemical and Molecular Probes

This strategy utilizes exogenous probes—such as chemical dyes, functionalized nanoparticles, or enzyme‐activatable substrates—to label bacteria. It does not necessarily require genetic modification of the bacteria, offering flexibility for clinical strains.

**FIGURE 6:**
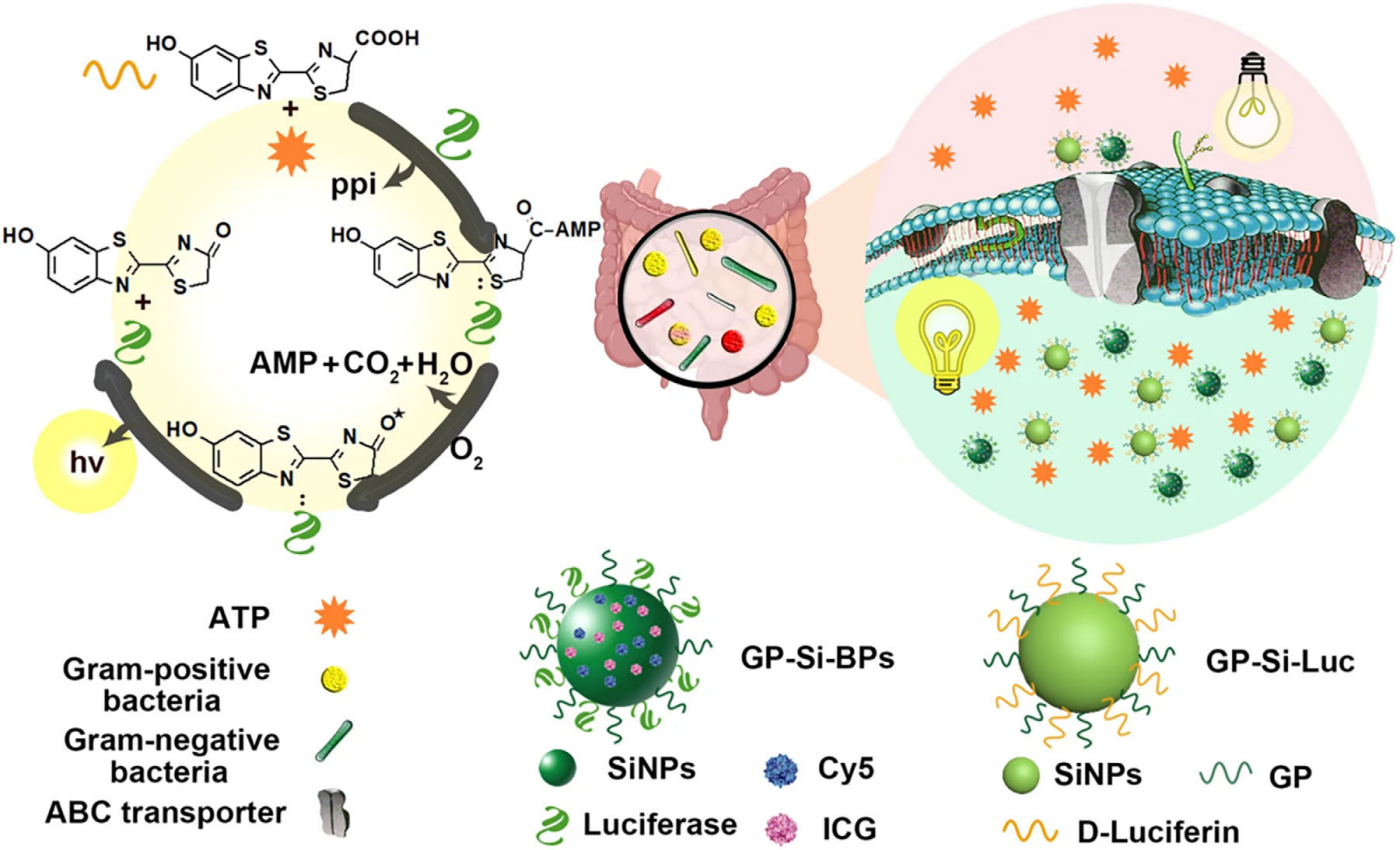
Schematic design of ABC sugar transporter enabling selective delivery of bioluminescent nanoprobes into Gram‐positive bacteria and Gram‐negative bacteria to visualize various natural bacteria in vivo with bioluminescence by directly consuming the ATP inside the bacteria [82]. The nanoprobes are made of GP, Cy5, ICG, and luciferase‐modified silicon nanoparticles (SiNPs) (GP‐Si‐BPs) and GP, D‐luciferin‐modified SiNPs (GP‐Si‐Luc). Reproduced with permission.[82] Copyright 2023, Springer Nature.

Some probes exploit unique bacterial transport mechanisms. Zhang et al. developed the Trojan BLI probe. This probe first utilizes the bacteria‐specific ATP‐binding cassette (ABC) sugar transporter to enable probe internalization by coupling luciferase and fluorescein to a unique bacterial carbon source. Subsequently, ATP activates bacterial fluorescein, initiates the reaction under luciferase catalysis, and further undergoes an energy transfer cascade via bioluminescence resonance energy transfer (BRET) between luciferase and Cy5, as well as fluorescence resonance energy transfer (FRET) between Cy5 and ICG. Finally, ICG generates near‐infrared signals capable of penetrating deep tissues, enabling imaging of viable bacteria in deep tissues (Figure 6) [82].

Probes can target bacterial surface structures (e.g., LPS) or leverage bacteriophage specificity. Zhang et al. developed a multifunctional live phage nanoconjugate (MS2‐DNA‐AIEgen bioconjugate) by integrating a luminescent agent (AIEgen) with aggregation‐induced emission properties and a nucleic acid molecule into a phage framework. These nanoconjugates can rapidly penetrate mammalian cells and accurately identify intracellular bacteria, leveraging the natural bacterial targeting of bacteriophages and the specific binding of nucleic acids. Particularly critical is the utilization of AIEgen's unique fluorescent properties: when specifically excited, they produce detectable fluorescent signals, enabling highly specific visual monitoring of bacteria [83]. Li et al. developed a polymyxin B‐fluorescein probe (PmBF) by conjugating fluorescein isothiocyanate (FITC) to the N‐terminus of polymyxin B (PmB). This probe can accurately target and label bacterial outer membrane vesicles (OMVs) by virtue of the specific binding of PmB to lipopolysaccharide (LPS) in the outer membrane of Gram‐negative bacteria. The fluorescent properties of FITC are then utilized to generate detectable fluorescent signals under specific excitation light. When combined with nanoflow cytometry, this enables high‐specificity quantitative analysis of OMVs in blood and early detection of in vivo bacterial infections [84].

Chen et al. have developed a novel near‐infrared fluorescent probe BIN‐3 for β‐lactamase. This probe comprises cephalosporin (a specific substrate for β‐lactamase), a near‐infrared HD fluorescent dye, hydrophilic zwitterionic groups, and carbamate leaving groups, and exhibits hydrophilic conversion and self‐immobilization properties. When the probe is inactive, it exhibits high water solubility due to its hydrophilic zwitterionic group. However, after hydrolysis by β‐lactamase, the hydrophilic group is removed, forming a hydrophobic quinone methide intermediate (QM). This intermediate effectively restricts probe diffusion and enhances bacterial uptake. Upon entering bacteria, QM covalently binds to nucleophilic groups within the bacteria, immobilizing fluorescent molecules therein and thereby generating persistent fluorescent signals at the detection sites. BIN‐3 is precisely targeted to and enriched in drug‐resistant bacteria via the specific substrate effect of cephalosporin on β‐lactamase. Once activated, it produces near‐infrared fluorescence, which can be detected upon irradiation with specific excitation light, enabling high‐specificity in vivo imaging of drug‐resistant bacterial infections [85].

### 4.2 Ultrasonic Imaging

Ultrasonic imaging is a noninvasive detection method based on the interaction between high‐frequency sound waves (1‐20 MHz) and biological tissues. It transmits sound waves, receives scattered signals reflected by tissue interfaces, converts echoes into electrical signals, and generates 2D/3D images after processing. Owing to its advantages of simple operation, strong real‐time performance, and absence of ionizing radiation, it is widely used in bacterial targeting and therapeutic efficacy evaluation for attenuated bacterial tumor therapy.

**FIGURE 7:**
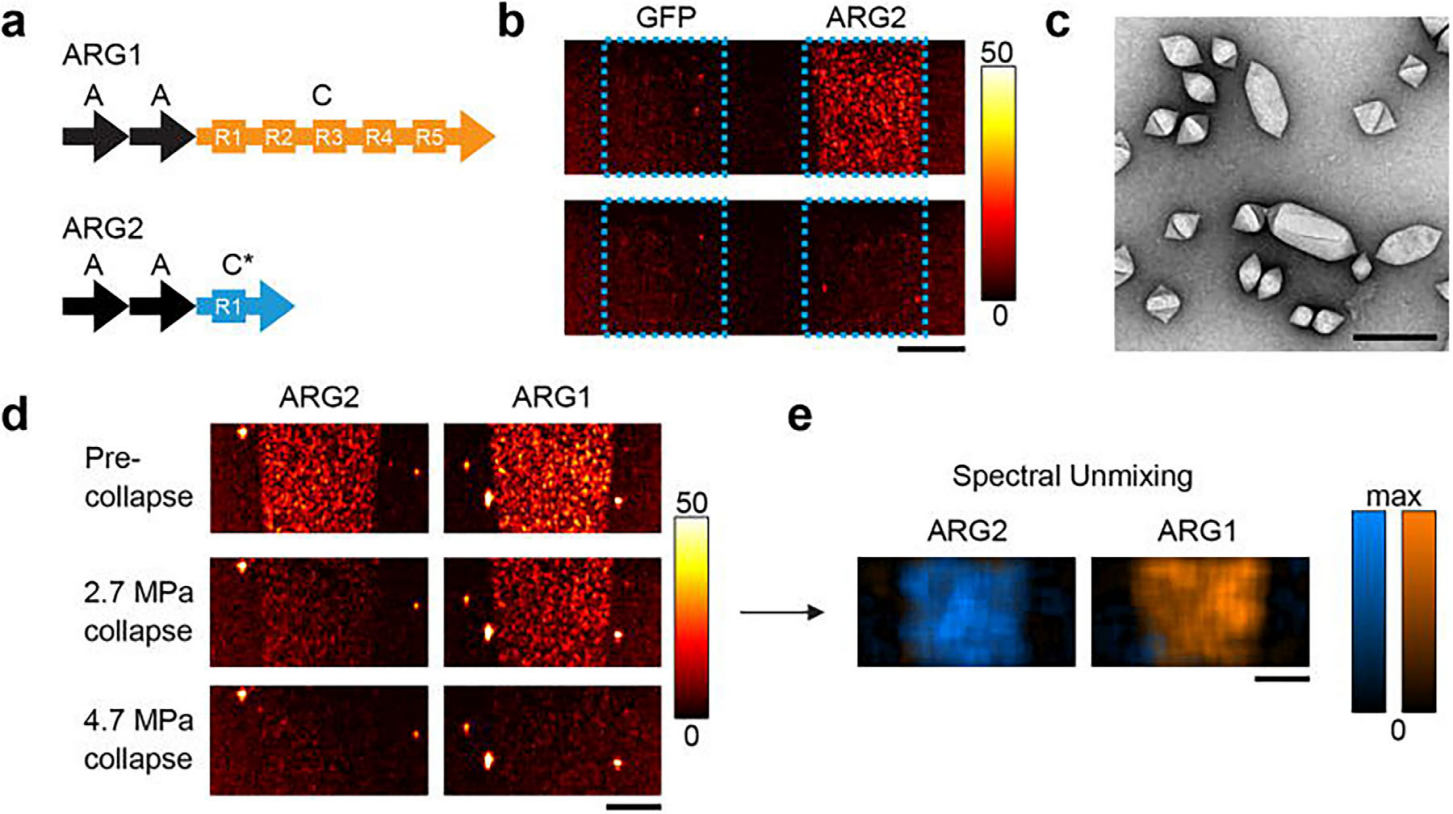
Multiplexed imaging of genetically engineered reporter variants [86]. (a) Diagram of the GvpA and GvpC sequences included in the ARG1 and ARG2 gene clusters. (b) Ultrasound images of a gel phantom containing *E. coli* expressing ARG2 or GFP (10^9^ cells/mL). Blue outlines indicate the location of each specimen. (c) Transmission electron micrographs of isolated ARG2 gas vesicles. (d) Ultrasound images of gel phantoms containing ARG1 or ARG2 before collapse, after collapse at 2.7 MPa and after collapse at 4.7 MPa (10^9^ cells / mL). (e) Overlay of the blue and orange‐colored maps from spectral unmixing of ARG2 and ARG1, based on the series of images in (d). Scale bars represent 2 mm in b), (d), (e), and 250 nm in (c). Each imaging experiment was repeated 3 times with similar results. Reproduced with permission.[86] Copyright 2018, Springer Nature.

Gas vesicles (GVs) are hollow proteinaceous nanostructures that scatter acoustic waves to generate ultrasound contrast signals. They can be collapsed by high‐voltage pulses to verify signal specificity. Acoustic reporter genes (ARGs) are genetically engineered components whose core is a modified gas vesicle gene cluster. This cluster enables bacteria to express GVs with acoustic properties, thereby enabling noninvasive visual monitoring of microorganisms via ultrasound imaging. Bourdeau et al. first constructed the gene cluster ARG1 by combining the GV gene cluster of Bacillus megaterium with the structural protein genes (gvpA, gvpC) of Anabaena. Its expression yielded larger and longer GVs and significantly enhanced ultrasonic scattering signals. Based on this, ARG2—with a lower GV collapse pressure—was obtained by truncating the gvpC gene. It enabled multiplex imaging with ARG1, thereby distinguishing different cell populations (Figure 7) [86].

Although ARGs have broad prospects, several issues remain, including low sensitivity, insufficient specificity, and poor long‐term stable expression of existing genes. Hurt et al. screened 288 GV‐expressing species and identified the superior GV gene cluster bARGser from Serratia sp. 39006. When expressed in *Escherichia coli*, it yielded a nonlinear ultrasound signal intensity ninefold higher than that of first‐generation ARGs. It could also be stably expressed at 37°C with a low metabolic burden [87].

Liu et al. developed an ultrasound‐responsive engineered bacterial delivery system (UEB) that enables specific tracking of bacterial functional activity via ultrasound‐induced heat‐triggered gene expression. It expresses catalase (CAT) and decomposes hydrogen peroxide exclusively in ultrasound‐irradiated regions, generating detectable functional signals [88]. Furthermore, when combined with focused ultrasound (FUS), it can penetrate deep tissues, act on deep tumors, and precisely activate intratumoral bacteria via thermal effects, thereby avoiding systemic exposure risks [89, 90].

### 4.3 Photoacoustic Imaging

Photoacoustic imaging (PAI) combines optical excitation and ultrasonic detection, converting the thermoelastic expansion of laser‐irradiated biological tissues into ultrasonic signals for imaging. This technology integrates the high contrast of optical imaging with the deep penetration of ultrasonic imaging, enabling accurate detection of physiological parameters.

Yang et al. designed responsive Cu_2_O nanoparticles (NPs) targeting hydrogen sulfide (H_2_S) and hydrogen peroxide (H_2_O_2_), which are highly expressed in bacterial infection microenvironments. Cu_9_S_8_ NPs, generated via the rapid reaction between Cu_2_O NPs and endogenous H_2_S, can act as near‐infrared window II (NIR‐II) photoacoustic contrast agents, accurately distinguishing inflammatory tissues from normal tissues. Based on the real‐time localization of infected areas by PAI, 1060 nm laser irradiation triggers the photothermal effect of Cu_9_S_8_ NPs, converting light energy into heat to generate local hyperthermia. Concurrently, these NPs can catalyze H_2_O_2_ to produce hydroxyl radicals, effectively killing bacteria and enabling specific photothermal treatment of infected sites [91].

Endogenous chromophores (e.g., hemoglobin, melanin) produce high background signals, limiting the sensitivity and specificity of tumor imaging. To address this, Gao et al. proposed the GPS imaging strategy: (G) a genetically engineered photochromic reporter protein (mDrBphP‐PCMm/F469W) that undergoes specific structural changes under light of different wavelengths, with a significantly increased absorbance difference. By switching between 780 nm/630 nm near‐infrared light irradiation, it can be clearly distinguished from background signals (which exhibit weak absorbance changes), thereby enhancing signal specificity in PAI; (S) combined with *Escherichia coli* expressing the photoswitch protein—utilizing their facultative anaerobic properties to specifically colonize and proliferate in the hypoxic tumor microenvironment—large quantities of the photoswitch protein are enriched in the tumor area, providing a sufficient signal source for subsequent PAI; (P) finally, background‐suppressed high‐resolution imaging of deep tissues is achieved via photoacoustic computed tomography (PACT) under 780 nm/630 nm near‐infrared light switching irradiation [92].

Zeng et al. developed engineered bacteria (CGB@ICG) by genetically incorporating ARGs and chemically modifying the bacterial surface with indocyanine green (ICG). This system can accurately colonize tumor tissues and be clearly visualized via the natural tumor‐targeting ability of *Escherichia coli* and the ultrasound imaging enhancement by GVs. Utilizing the near‐infrared fluorescent properties of ICG, when excited by an 808 nm laser, it produces detectable fluorescent signals that enable dynamic tracking of bacterial accumulation. Additionally, ICG supports PAI, with its signal linearly correlated with bacterial concentration, aiding in the visualization of bacterial distribution. This multimodal imaging integrates the advantages of different imaging modalities to accurately monitor the colonization, distribution, and accumulation of CGB@ICG in tumors. It facilitates real‐time monitoring and evaluation of clinical therapeutic effects, ensuring stable efficacy [93].

### 4.4 Magnetic Resonance Imaging

Magnetic resonance imaging (MRI) is an important tool that uses specific markers to distinguish bacteria from surrounding tissues. It enables noninvasive visual monitoring of engineered bacteria through its high soft tissue resolution and deep penetration capability, and facilitates the study of bacterial distribution in complex organisms.

Bacterial magnetosomes (BMs) are natural magnetic nanomaterials synthesized by magnetotactic bacteria (e.g., Magnetospirum gryphiswaldense MSR‐1). They consist of magnetite crystals enclosed in a lipid bilayer membrane and possess advantages such as uniform size, high crystallinity, good biocompatibility, and ease of surface modification, making them highly promising in the field of MRI [94].

**FIGURE 8:**
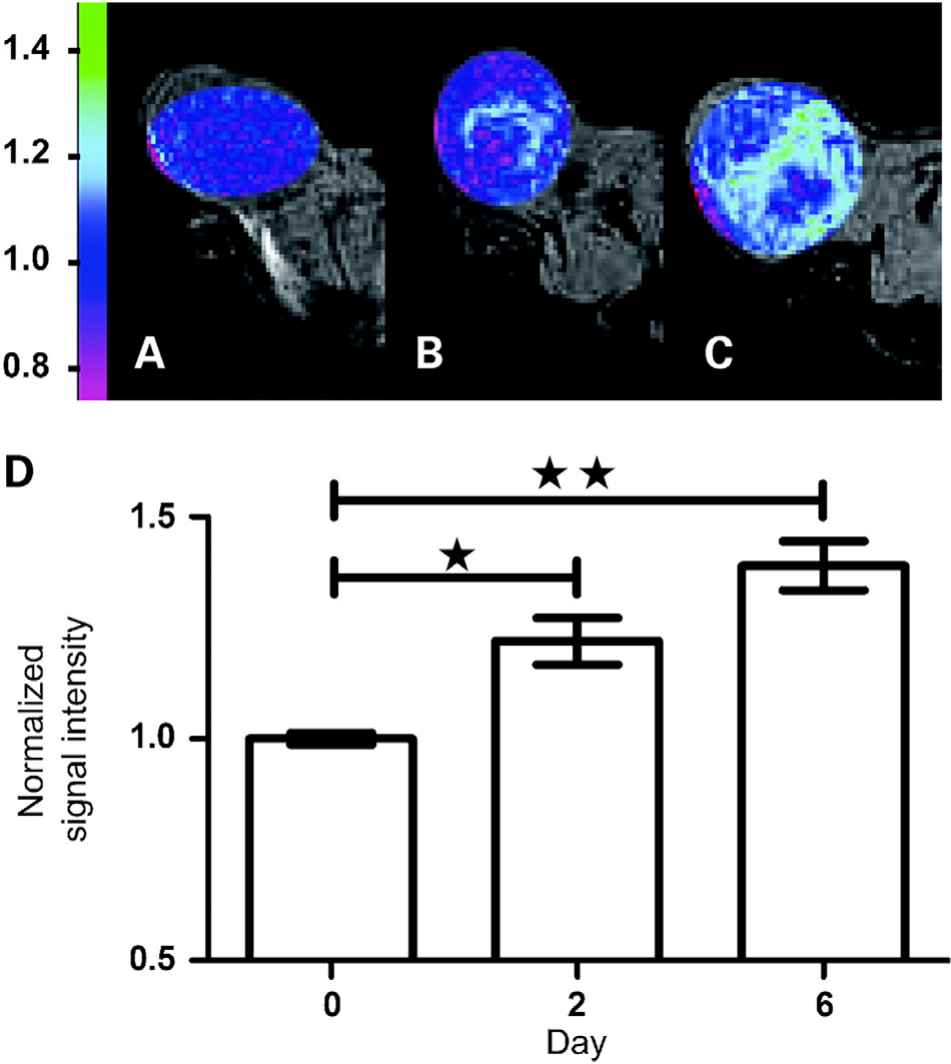
MRI of magnetotactic bacteria AMB‐1 in a tumor xenograft model following tail vein injection [95]. (A) Representative T1‐weighted axial MRI scan of tumor tissue prior to injection (Day 0). (B) Corresponding MRI scan of the same tumor acquired 2 days post‐injection (Day 2). (C) MRI scan acquired 6 days post‐injection (Day 6). (D) Quantitative analysis of normalized signal intensity within the tumor region. Data are presented as mean ± s.e.m. (*n* = 4). Statistical significance was determined using a paired Student's *t*‐test. *P* = 0.003, *P* = 0.0007. Reproduced with permission [95]. Copyright 2009, American Association for Cancer Research.

Benoit et al. were the first to demonstrate that the magnetotactic bacterium AMB‐1 can alter MRI contrast properties by modulating magnetite particle size. In bacterial cultures, when FeCl_3_ is used as the iron source, the bacteria produce ∼25 nm small particles that generate T1‐weighted positive contrast (signal enhancement); when iron malate is used as the iron source, ∼49 nm large particles are produced, which mainly generate T2‐weighted negative contrast (signal attenuation). Following systemic administration, AMB‐1 specifically colonizes tumors, and MRI signal enhancement correlates with bacterial colonization and survival in tumors, enabling noninvasive tumor imaging (Figure 8) [95].

However, as a medical contrast agent, its long‐term distribution, clearance pathways, and biological safety after in vivo administration remain unclear. Nan et al. clearly monitored the distribution of BMs in the liver, spleen, and other deep organs through a 135‐day long‐term follow‐up. They confirmed that BMs exhibit good biocompatibility via a dual‐channel clearance process (fecal and urinary) over time, providing key safety data for clinical translation [94]. However, the synthesis of magnetosomes by AMB‐1 depends on its intrinsic natural mechanism, with signal stability being difficult to control and functional expansion being limited, thus requiring further research and exploration. Mms6 is a protein closely involved in magnetite biosynthesis in magnetotactic bacteria. Yavuz et al. successfully constructed engineered *Escherichia coli* capable of intracellular/extracellular magnetite accumulation via genetic engineering. ECMAB expresses a fusion of Mms6 and the autotransporter protein Ag43 on the cell surface and synthesizes magnetite nanoparticles there; ICMAB expresses a combination protein: the iron transporter EfeU promotes ferrous ion uptake, ferroxidase oxidizes it to ferric iron, and Mms6 and the material‐binding peptide MBP cooperatively mediate intracellular magnetite synthesis and accumulation. Both types of engineered bacteria can serve as high‐efficiency MRI contrast agents, enabling noninvasive monitoring of bacteria in organisms via MRI's high resolution and deep penetration capabilities [96]. In the future, this system can be transplanted into attenuated bacterial strains to enable accurate and controllable detection in tumor therapy.

Donnelly et al. studied longitudinal and transverse MRI relaxation rates and demonstrated that the inherent metal content of bacteria can serve as an endogenous MRI contrast agent. It exhibits strain specificity (e.g., Lactobacillus has high manganese and low iron, with a relaxation rate much higher than that of other bacteria) and correlates positively with the proportion of viable bacteria, thus reflecting bacterial activity. Without exogenous labels, it can distinguish specific microorganisms in complex flora in real time, providing a real‐time visualization tool for studying host‐microorganism interactions and the dynamic distribution of flora—particularly for microbial research involving unculturable organisms [97].

### 4.5 Raman Spectroscopy

Raman spectroscopy (RS) is a label‐free imaging technique that combines spectroscopy and microscopy, based on the inelastic scattering effect (Raman scattering) of photons interacting with matter. By scanning the sample point‐by‐point, collecting Raman spectra at each point, and integrating chemometric analysis, high‐resolution images containing spatial distribution and chemical composition information are generated.

Surface‐enhanced Raman spectroscopy (SERS) is a spectroscopic analysis technique based on the Raman scattering effect. It enables the detection of trace substances by significantly enhancing the Raman signals of molecules adsorbed on or near the surface of metal nanostructures (e.g., gold and silver nanoparticles). For example, Liu et al. proposed a WPGA algorithm based on Wavelet Packet Transform and Gramian Angular Field (GAF). This algorithm compresses 1D SERS data by 15‐fold, converts it into 2D spectra, and enables rapid bacterial identification by integrating a convolutional neural network (CNN) [98]. Different bacterial species have distinct compositions, which are reflected in Raman spectra as unique characteristic peaks. Liu et al. prepared acetic acid‐treated silver nanorod (AgNR)‐based SERS substrates via an interfacial self‐assembly method. When laser light irradiates the substrates, strong electromagnetic fields are generated near the nanorod surfaces. After bacteria are adsorbed onto the substrate surface, the Raman scattering signals of their intrinsic molecules are significantly amplified under this enhanced electromagnetic field  . This renders the originally weak Raman signals easily detectable, enabling the acquisition of clearer bacterial information, rapid and specific identification of 22 pathogenic bacterial species, and laying a foundation for the automatic on‐site detection of pathogenic bacteria in complex scenarios [99] (Table 3).

**TABLE 3:** Comparison of In Vivo Bacterial Detection and Imaging Technologies.

Modality	Strategy	Key Mechanism	Key Features
Optical Imaging	Genetically Encoded	Endogenous expression of optical proteins: Utilization of bacterial transcriptional and translational machinery to express luciferase (enzymatic bioluminescence) or fluorescent proteins/tags (photoexcitation or ligand‐binding luminescence).	Facilitates real‐time monitoring of bacterial viability and proliferation as signals are inherited during cell division; ATP‐dependent enzymatic luminescence (e.g., *Lux*) serves as an indicator of bacterial metabolic activity.
	Exogenous Probes	Exogenous targeted recognition: Activation of exogenous fluorescent probes via bacterial‐specific transporters (BRET/FRET), surface structures (LPS/OMVs), or enzymes (e.g., β‐lactamase).	Suitable for non‐engineered clinical or drug‐resistant strains with significantly reduced background noise; signal intensity and specificity depend on the delivery and penetration efficiency of probes within the tumor site.
Ultrasound Imaging	Genetically Encoded	Bacterial expression of hollow protein nanostructures (gas vesicles) to scatter acoustic waves, or expression of specific enzymes to catalyze metabolites into oxygen microbubbles for echo enhancement.	High deep‐tissue penetration without the risk of ionizing radiation; distinct acoustic collapse pressures of different gas vesicles enable multiplexed signaling; allows visualization of bacterial gene expression in deep tumor tissues.
Photoacoustic Imaging	Hybrid	Bacterial uptake or surface modification with nanomaterials possessing high photothermal conversion efficiency (e.g., gold nanoparticles, copper sulfide), generating ultrasonic signals via thermal expansion upon laser irradiation.	Combines the high contrast of optical imaging with the deep penetration of ultrasound; excitation wavelengths can be tuned to the NIR‐II window to minimize biological tissue interference; often integrates photothermal therapy (PTT) for theranostic applications.
MRI	Genetic/Endogenous	Intracellular biosynthesis of membrane‐bound magnetic crystals or natural enrichment of paramagnetic metal ions (e.g., manganese), thereby interfering with proton spin relaxation.	Provides high‐resolution anatomical images of soft tissues; biosynthesized crystals exhibit uniform nanosize and excellent biocompatibility; bacterial localization is achieved via T1 (brightening) or T2 (darkening) signal alterations.
	Exogenous Probes	Paramagnetic probes undergo cleavage by bacterial‐specific enzymes (e.g., gelatinase), leading to hydrophobic shifts and self‐assembly on the bacterial surface.	Highly flexible design allows for “smart” responsive imaging tailored to specific microenvironments or antibiotic‐resistance enzymes.
Raman Spectroscopy	Exogenous Assisted	Surface‐Enhanced Raman Scattering (SERS): Enhancement of inelastic light scattering signals from bacterial molecules using metallic nanostructures (e.g., silver nanorods).	Label‐free detection; provides “fingerprint” spectroscopic information reflecting bacterial biochemical compositions, enabling precise identification of bacterial species.

## 5 Bacterial Industrial Production

Attenuated bacterial tumor therapy is currently an important research direction. However, compared with traditional small‐molecule drugs or antibody‐based drugs, the production of live Bacterial drugs is more complex. There are numerous challenges in transitioning from laboratory research to industrial production, including key steps such as strain preservation, large‐scale fermentation processes, downstream processing technologies, formulation stability, and transportation management.

### 5.1 Culture Preservation

Culture preservation and passage is the first step in the industrial production of attenuated bacteria for tumor therapy. During long‐term passage, engineered strains may undergo plasmid loss, gene mutation, or virulence reversion, which directly affects product quality and efficacy. Therefore, maintaining the genetic stability of engineered strains is critical for ensuring therapeutic efficacy and safety.

Currently, common industrial methods for culture preservation mainly include agar slant preservation, mineral oil immersion, carrier drying, and low‐temperature freezing, among others.

The agar slant method provides a larger surface area and stable growth environment for microorganisms by solidifying agar medium in inclined test tubes to form a slant. Its core principle is to utilize the solidification properties of agar (typically 1.5%–2.0%) to form a solid support matrix, which combines with nutrients to promote the proliferation, preservation, or expression of biochemical characteristics of target microorganisms. Hébert et al. verified the preservation effect of charcoal‐yeast extract agar slant medium on different Legionella strains under room temperature and dark conditions, providing a reference for laboratory strain preservation [100]. This method is simple and low‐cost but requires frequent subculturing and is prone to mutation [101]. It is suitable for short‐term preservation and maintenance of working strains.

To extend preservation duration, mineral oil (liquid paraffin) can isolate air, reduce oxygen permeability, induce microorganisms to enter a dormant state, and absorb microbial metabolites (e.g., organic acids) to delay pH changes in the medium. Mineral oil immersion is not suitable for short‐term (1–2 years) preservation; however, some strains may utilize paraffin as a carbon source, so its applicability needs to be tested in advance [102]. Alternatively, the carrier drying method uses porous media such as sand, silica gel, and activated carbon as carriers. These materials have a high specific surface area and strong water absorption capacity, enabling them to quickly absorb free water in biological samples. During dehydration, bacteria enter a dormant state, the metabolism of bioactive substances almost ceases, and long‐term preservation is achieved [103]. For spore‐producing microorganisms (e.g., Clostridium spp.), carrier drying methods (e.g., sand preservation) exhibit better stability, with a shelf life of 1–10 years.

Cryopreservation technology has become the preferred method for constructing industrial strain banks due to its advantages of long‐term preservation, maximal genetic stability, and reduced mutation accumulation during subculturing. The core of bacterial cryopreservation is to achieve long‐term preservation by inhibiting intracellular enzyme activity and metabolism at low temperatures. Commonly used methods mainly include ultra‐low temperature freezing (−80°C) and liquid nitrogen freezing (−196°C). Cryopreservation is easy to operate and low in cost, making it suitable for short‐to medium‐term preservation of most bacteria (2–5 years). Liquid nitrogen freezing can achieve a longer‐term preservation effect (>10 years) and is particularly suitable as a preservation method for master strains.

Cryoprotectants [104] are primarily used to reduce tissue damage during freezing (cooling) and resuscitation (warming). Glycerol (10%–15%) and dimethyl sulfoxide (DMSO; 5%–10%) are the most commonly used cryoprotectants [105, 106]. Glycerol is mostly used for Gram‐negative bacteria such as *Escherichia coli*, and its protective mechanism primarily involves maintaining osmotic pressure balance between the intracellular and extracellular environments through osmotic regulation. In contrast, DMSO can penetrate the cell membrane and directly protect intracellular structures [107]. For some sensitive engineered strains, composite protectants (e.g., glycerol + DMSO + trehalose) often provide better protection. Research by the China Institute for Food and Drug Control shows that the optimized composite protectant formula can increase the cryopreservation survival rate of some attenuated Salmonella strains from 60% to over 90%.

In industrial practice, it is standard to establish a three‐level strain bank management system (master generation, working generation, and production generation) to ensure genetic stability. Master generation strains are typically stored via freeze‐drying; working generation strains are stored in liquid nitrogen or at −80°C; and production generation strains are either stored via short‐term refrigeration or freezing or appropriately amplified based on production scale. Strict genetic stability verification is required for each generation of strains, including restriction enzyme analysis, whole‐genome sequencing, and functional activity testing. There are significant differences in the cryopreservation adaptability among different strains. For example, due to their cell wall structural characteristics, some Gram‐positive bacteria typically tolerate the cryopreservation process better than Gram‐negative bacteria [108, 109]; the cryopreservation survival rate of spore‐forming bacteria is often higher than that of non‐spore‐forming bacteria [110, 111]. This indicates that cryopreservation stability factors should be considered during strain selection.

In recent years, with the rapid development of materials science and biotechnology, several innovative materials and strategies have been proposed to improve the efficiency and quality of biological sample cryopreservation. New cryopreservation materials include carbohydrates, polymers, antifreeze proteins (AFPs), ice‐nucleating agents, and hydrogels, among others. These materials improve cell survival and recovery by inhibiting ice crystal recrystallization, reducing intracellular ice crystal formation, and suppressing supercooling [112]. Nanoscale heating, cell encapsulation, freezing grids, and isovolumetric freezing, among other methods, are gradually enabling large‐scale cryopreservation [112].

### 5.2 Large‐Scale Culture

Large‐scale bacterial culture constitutes a critical step in achieving industrialization [113]. In contrast to laboratory‐scale cultivation, industrial‐scale culture must address core challenges including high‐density cultivation, metabolic regulation, and process consistency—factors that directly influence product yield, quality, and production costs.

Optimization of the culture medium forms the foundation for the advancement of large‐scale cultivation. Diverse bacterial species exhibit distinct nutritional requirements. The selection of carbon and nitrogen sources not only influences bacterial growth rates but also alters the expression levels of related proteins. For instance, the optimal carbon and nitrogen sources for *Escherichia coli* BL21 consist of 40 mM glycerol combined with 10 g/L tryptone [114], for Bacillus subtilis YPS‐32, they are 20 g/L molasses, 15 g/L glutamic acid, and 4.5 g/L soybean meal [115], and for Streptomyces 891‐B6, they include 39.283 g/L glucose, 15.480 g/L soybean meal, with 20.662 g/L corn starch serving as an auxiliary carbon source [116].

Genetically engineered bacteria typically harbor foreign plasmids, which may be lost during bacterial division due to uneven distribution or structural instability, leading to a loss of therapeutic function. To ensure the genetic stability of engineered bacteria during the production process, a rational selection pressure strategy must be employed. While the addition of antibiotics is one of the most common methods utilized in laboratories, it is impractical for industrial‐scale applications due to cost and ecological constraints [117].

Antibiotic‐free plasmid maintenance systems utilize forward synthetic biology design to address the challenge of plasmid loss. Brechun et al. developed an antibiotic‐free plasmid fermentation method by genetically modifying the essential gene infA of E. coli, placing it under an arabinose‐inducible promoter, and inserting a copy of the gene into the plasmid. In the absence of arabinose, cells can only survive via plasmids harboring infA, thereby maintaining the plasmids; conversely, the addition of arabinose enables strains to proliferate without plasmids. The results demonstrated that the modified strain could maintain 100% plasmid retention and exhibit faster growth, thereby avoiding the risk of antibiotic resistance [118]. Furthermore, we have included another effective strategy utilizing Toxin‐Antitoxin (TA) systems. In plasmid‐bearing cells, the plasmid encodes a stable toxin alongside a labile antitoxin that neutralizes its toxicity. If a bacterium loses the plasmid during division, the rapidly degraded antitoxin cannot be replenished, allowing the persistent toxin to exert its lethal effect and specifically eliminate plasmid‐free progeny [119]. The classical Type II TA system (e.g., the Axe/Txe system) operates through this mechanism and has demonstrated superior performance in stabilizing therapeutic plasmids; consequently, it has been cataloged as a standardized tool in synthetic biology [120]. These two strategies provide a robust technical framework for maintaining plasmid stability. When integrated with rigorous, industry‐grade production quality control systems, they establish a comprehensive safeguard for transitioning from fundamental research to clinical translation.

To control mutations or plasmid loss induced by environmental stressors during the transition from in vitro to in vivo conditions, at the bioprocessing level, the core strategy shifts from reactive management of environmental stressors to proactive pre‐adaptation and selection under simulated in vivo conditions. During the process development phase, conditions mirroring the tumor microenvironment—such as hypoxia, acidic pH, or nutrient limitation—can be applied during fermentation to directionally select high‐fitness clones that maintain genetic stability and functional integrity under such pressures [121]. Concurrently, the implementation of Process Analytical Technology (PAT) enables a Quality‐by‐Design (QbD) approach. By leveraging advanced inline sensing and data analytics, key process parameters (KPPs)—including pH, dissolved oxygen, and metabolite concentrations—are monitored in real‐time. This allows for the dynamic design, analysis, and control of the manufacturing process rather than relying solely on end‐product testing. Consequently, this integrated approach ensures batch‐to‐batch consistency and mitigates genetic drift induced by unforeseen environmental stressors [122].

The choice of culture mode constitutes a critical consideration in process development. Traditional batch culture entails inoculating microorganisms into a pre‐prepared medium in a single batch, with the entire cultivation process (from inoculation to harvest) conducted within a closed system. DDuring this period, no additional nutrients are supplemented, and no culture broth is discharged. This method is easy to operate but exhibits low efficiency due to substrate depletion, accumulation of inhibitory metabolites, and the requirement for equipment preparation in each cycle [123, 124]. Fed‐batch culture is based on batch culture, with nutrients (e.g., carbon sources, nitrogen sources, and trace elements) added in stages or continuously according to bacterial growth requirements, thereby avoiding nutrient limitation or metabolite inhibition [125, 126]. Continuous culture operates in an open system, where fresh medium is continuously introduced into the cultivation tank, with an equal volume of culture broth discharged at the same rate. This maintains stable nutrient concentrations, cell densities, and metabolic states within the cultivation system, enabling theoretically long‐term operation. However, it imposes extremely high demands on operational control and contamination prevention [124, 127].

Dissolved oxygen levels represent a critical parameter for the cultivation of aerobic bacteria [128]. In large‐scale cultivation, maintaining adequate dissolved oxygen levels while avoiding excessive shear stress that may damage bacteria presents a major challenge [129]. The novel airlift fermenter is a pneumatic fermentation device without mechanical agitation, which accomplishes liquid mixing and gas transfer via the injection and circulation of gases (e.g., air or oxygen) [130, 131]. The membrane aeration system enables efficient dissolution of gases (e.g., oxygen) or removal of gases (e.g., carbon dioxide) via a semi‐permeable membrane, while reducing bubble generation, energy consumption, and shear force [132, 133]. Both systems provide a gentler mode of oxygen transfer, rendering them suitable for shear‐sensitive bacteria.

For the industrial cultivation of anaerobic bacteria, common cultivation containers include Hungate tubes, Balch tubes, and serum bottles. These can be sealed with butyl rubber stoppers and aluminum caps, and pre‐filled with matrix gas to establish a suitable gaseous environment. For large‐scale cultivation, bottles with sealable necks or pressure‐tolerant Schott bottles can be employed. These containers have large volumes and can be connected to gas pipelines via interfaces for substrate gas replacement or supplementation, satisfying both the requirements of large‐scale cultivation and the controllability of the gaseous environment [134]. Furthermore, the development of oxygen removal measures is particularly crucial. Wang et al. developed a novel electrochemical oxygen removal (EOR) controller featuring a self‐powered design. It utilizes a sacrificial anode to drive the cathodic oxygen reduction reaction (ORR), efficiently consuming oxygen from the environment without requiring external gas or power. Moreover, the system incorporates electronic components that can start and stop the oxygen removal process to precisely regulate the target oxygen concentration and adjust the current to control the oxygen removal rate. This overcomes the limitations of traditional oxygen removal methods (e.g., high nitrogen purge costs and poor controllability of chemical oxygen absorption) and exhibits broad application potential [135].

Effective monitoring and control of biological processes are pivotal for industrial biomanufacturing. Xu et al. integrated near‐infrared and RS data via machine learning algorithms (e.g., ridge regression, gradient boosting, and multilayer perceptron), facilitating the development of predictive and adaptive fed‐batch fermentation strategies [136]. This intelligent control method is particularly suitable for attenuated bacteria sensitive to cultivation conditions, as it can dynamically adjust dissolved oxygen levels, pH, and feeding strategies to balance the relationship between bacterial growth and the accumulation of therapeutic products.

### 5.3 Downstream Processing

Following fermentation, the efficient recovery of bacteria and preparation of stable formulations represent another critical step in the industrial production of attenuated bacteria for tumor therapy. Downstream processes include cell separation, washing, and drying, each of which can impact the quality and activity of the final product.

Centrifugal separation is the most widely used method for bacterial recovery. Industrially, disc centrifuges are utilized, with processing capacities of several cubic meters per hour [137, 138]. Wang et al. utilized an automated centrifugation system to enrich target bacteria via immunomagnetic separation (IMS) combined with recombinant enzyme‐assisted amplification (RAA). Under optimal conditions, the detection limit for Salmonella in spiked chicken samples was as low as 10 CFU/mL, with a mean recovery rate of 105.6% and a mean standard deviation of 8.4%, demonstrating its potential as an alternative method for rapid Salmonella detection [139]. Traditional centrifugal pretreatment for large‐volume (≥1 L) bacterial cultures encounters challenges such as limited equipment capacity, time consumption, and potential bacterial damage. Allahghadry et al. developed a centrifugation‐free clarification scheme for large‐volume bacterial cultures, using *Escherichia coli* with high yields of OMVs as a model. By adding 80 µg/mL of diatomaceous earth (DE) to the bacterial culture, employing a polyethersulfone (PES) filter membrane with a 0.22 µm pore size and 76 cm^2^ filter area, and combining it with a high‐intensity vacuum pump, rapid clarification was achieved without centrifugal pretreatment, while maximizing retention of the target products. This offers a practical alternative for the extraction of industrial‐grade microbial products, particularly for the separation and purification of relatively fragile bacteria [140].

The washing step is designed to remove medium residues and metabolic byproducts. Concentration prior to washing reduces buffer consumption and improves efficiency. Traditional precipitation methods, e.g., ammonium sulfate precipitation, have drawbacks: high salt concentrations can damage bacteria via osmotic pressure imbalance, disrupt cell structures, and interfere with their metabolic environment. Furthermore, the non‐specificity of precipitation can easily complicate bacterial recovery, and subsequent desalination steps may further impair the activity of viable bacteria [141, 142]. In contrast, ultrafiltration concentration allows small molecular substances to permeate membrane pores via pressure‐driven processes without introducing chemical reagents, enabling effective concentration while preserving bacterial activity [143, 144]. Washing buffers must be carefully selected—typically saline or specially formulated buffers—to prevent bacterial damage caused by osmotic shock.

Drying is critical for ensuring the long‐term stability and therapeutic efficacy of products. Currently, freeze‐drying (lyophilization) and spray drying are the primary methods employed in industry. Spray drying operates by dispersing bacterial suspensions into tiny droplets via an atomizer, which are then rapidly dried upon contact with hot air. This method features low energy consumption and high continuity, rendering it a more economical option for certain heat‐resistant spore‐forming bacteria [145]. Freeze‐drying, owing to its mild dehydration characteristics, is the most widely used drying method for live bacterial drugs. The technique involves freezing the sample and then sublimating ice directly under vacuum, thereby avoiding structural damage induced by liquid water.

Despite its advantages, freeze‐drying can still cause bacterial membrane damage and protein denaturation due to ice crystal formation and dehydration stress. An effective approach to mitigate this is to optimize the formulation of freeze‐drying protectants. For example, Yuan et al. mixed skimmed milk, trehalose, and inulin with Lactobacillus plantarum W1 at a mass ratio of 1:8, resulting in a freeze‐drying survival rate of 86.87% [146]. Tian et al. identified optimal freeze‐drying protectant combinations for specific strains: for Lactobacillus BZ11, 10% skimmed milk powder, 3% sodium glutamate, and 15% trehalose; for Lactobacillus plantarum LB12, 10% skimmed milk powder, 5% sodium glutamate, and 10% trehalose; and for Streptococcus thermophilus Q‐1, 10% skimmed milk powder, 3% sodium glutamate, and 10% trehalose [147]. Jayaprakash et al. compared the dehydration effects of electrostatic spray drying, traditional spray drying, and freeze‐drying on Lactobacillus rhamnosus GG. Jayaprakash et al. compared the dehydration effects of electrostatic spray drying, traditional spray drying, and freeze‐drying on Lactobacillus rhamnosus GG. It thus emerges as a promising and scalable alternative, particularly for heat‐sensitive probiotic therapies (with skim milk as the optimal embedding agent) [148].

### 5.4 Formulation and Transportation

In the development of formulation processes, comprehensive consideration must be given to bacterial stability, administration routes, and clinical safety. For oral formulations, acid‐induced degradation and intestinal competitive colonization present the greatest challenges. Encapsulation within alginate‐gelatin‐chitosan (APC) microcapsules significantly improves the survival of Pediococcus lactis in simulated gastric and intestinal fluids, outperforming unencapsulated strains and enhancing therapeutic efficacy in vivo [149]. Microencapsulation technology can not only protect bacteria from the gastric acid environment but also enable targeted drug delivery [150]. Wang et al. developed a core‐shell structured microcapsule (RSV@ALG‐CS) fabricated via microfluidic electrospray technology. The outer chitosan hydrogel degrades gradually in the acidic environment (pH 5.4) of infected wounds, enabling sustained and precise release of internal resveratrol (RSV)—a finding that provides insights for targeting the tumor microenvironment [151]. Injectable formulations require greater caution to mitigate the risk of systemic infection [152]. Chen et al. mixed engineered Lactococcus lactis (FOLactis) with the temperature‐sensitive hydrogel poloxamer 407 (P407), which is liquid at room temperature (facilitating injection) and rapidly forms a gel at body temperature, achieving local sustained release and prolonged tumor retention [153].

Viable bacterial drugs are far more sensitive to environmental conditions (e.g., temperature, humidity, oxygen) than traditional drugs, thereby imposing specific requirements on the management of product storage and transportation. Most live Bacterial drugs require cold chain transportation (2°C–8°C) to maintain bacterial viability [154]. However, cold chain management not only increases logistics costs but also creates barriers to utilization in regions with inadequate infrastructure. To address this issue, the development of room‐temperature‐stable formulations represents an important research direction. Bajrovic et al. developed a novel thin‐film technology. By screening over 400 formulations, an optimized thin‐film matrix was developed, consisting of a substrate, a binder (e.g., sorbitol), and a surfactant. Its core lies in forming an amorphous solid structure with a glass transition temperature higher than room temperature, which restricts molecular movement and preserves the structural integrity of biopharmaceuticals via nitrogen‐hydrogen bonds. Live bacteria encapsulated in this film can be stored at room temperature for 8 months without cold chain support, significantly lowering storage and distribution costs. This advancement improves global access to biopharmaceuticals, particularly in resource‐poor regions [155].

## 6 Bacterial Marketing Authorization

The uniqueness of live biopharmaceuticals lies in their active ingredients being living microorganisms, which makes it challenging to directly apply traditional CMC (Chemistry, Manufacturing and Control) standards designed for chemical drugs. Key parameters of live biopharmaceuticals, e.g., potency and colonization capacity, are difficult to quantify, creating obstacles for regulatory authorities in assessing their consistency and stability [156]. With the rapid advancement of clinical research on bacteriotherapy, global regulatory authorities are actively developing review frameworks to accommodate these new therapeutic products, aiming to balance the acceleration of innovation and the mitigation of risks.

### 6.1 Standard for Approval of Viable Drugs

Regulatory authorities in different countries and regions have significant differences in their approval requirements for live bacterial drugs. In China, according to the 1999 Measures for the Approval of New Biological Products, microbial preparations are explicitly classified as biological products, which dictates that their R&D must comply with GLP (Good Laboratory Practice) and production must adhere to GMP (Good Manufacturing Practice). Additionally, the Measures for the Administration of Drug Registration set forth more specific requirements for live bacterial drugs on this basis. According to Article 13 of these Measures, applicants shall provide comprehensive and reliable research data to demonstrate the safety, efficacy, and quality controllability of the drugs, and shall be responsible for the authenticity of all data.

In terms of specific technical requirements, for live bacterial drugs, enterprises shall submit complete strain identification data in accordance with Article 21, covering microbial taxonomic characteristics and gene sequence analysis, to ensure the traceability and specificity of strains. Additionally, data on genetic stability research shall be provided to demonstrate the absence of harmful mutations in key genes during strain passage, thereby preventing fluctuations in efficacy or safety risks. Regarding in vivo biodistribution characteristics, enterprises are required to conduct relevant research to clarify the colonization location, quantity, and duration of bacteria in host organs and tissues, in order to understand their targeting and potential spread risks. Furthermore, a long‐term safety assessment report is also essential. According to Article 121, within the validity period of the drug approval number, Import Drug Registration Certificate, or Pharmaceutical Product Registration Certificate, the applicant shall systematically evaluate the safety, efficacy, and quality control of the drug, including relevant research results, adverse reaction monitoring, production control, and product quality uniformity during the monitoring period. Animal experiments and early clinical studies should be conducted to assess potential adverse reactions, such as immune responses and infection risks, caused by long‐term administration. These requirements are closely interlinked, ranging from classification definitions to specific technical standards, and form a full‐chain supervision system. The ultimate goal is to ensure that live bacterial drugs possess both safety and efficacy under the premise of controllable quality, and to establish a legal guarantee for clinical application.

In Early Clinical Trials with Live Biotherapeutical Products: Chemistry, Manufacturing, and Control Information, the US Food and Drug Administration (FDA) has established differentiated regulatory pathways for various types of bacterial therapeutic products, including single‐bacterial preparations, multi‐bacterial combination preparations, and fecal bacterial transplant products. The guidelines specifically emphasize several critical control points: first, strict donor screening criteria must be established; second, comprehensive pathogen testing procedures need to be implemented; and finally, strict control over the manufacturing process must be maintained [157, 158]. These requirements reflect the FDA's concerns regarding the specific risk factors of viable biological products and provide clear regulatory guidance for the development of such innovative products.

### 6.2 Reasons for Difficulties in Approving Viable Drugs

In recent years, living therapeutics have demonstrated immense promise in the field of cancer immunotherapy, with oncolytic viruses and oncolytic bacteria emerging as the two primary therapeutic platforms. As the only “living” cancer therapies currently granted FDA marketing approval (e.g., T‐VEC, H101), oncolytic viruses have successfully established the clinical viability of replication‐competent biologics in oncology. Distinct from prophylactic vaccines, both oncolytic bacteria and oncolytic viruses must maintain their “living” state to exert therapeutic efficacy: viruses require active infection and lysis of tumor cells, whereas bacteria must actively migrate to and colonize the deep tumor parenchyma. While inactivation treatments ensure the safety of traditional vaccines, such processes would completely abolish the therapeutic activity of these oncolytic agents.

Despite their shared classification as “living medicines,” oncolytic bacteria currently lag behind oncolytic viruses in terms of clinical translation progress. However, this discrepancy does not imply a lack of potential in bacterial platforms. On the contrary, oncolytic bacteria exhibit unique advantages over viral vectors in areas such as administration routes, genetic payload capacity, deep‐tissue penetration, and safety control mechanisms—factors that serve as the core drivers for the continued advancement of this field. We provide a detailed comparison of the characteristics, strengths, and limitations of these two therapeutic modalities in Table 4. Comparative Analysis of Oncolytic Viruses and Oncolytic Bacteria.

**TABLE 4:** Comparative Analysis of Oncolytic Viruses and Oncolytic Bacteria.

Feature	Oncolytic Viruses	Oncolytic Bacteria
Structure and Size	Structurally simple (nanoscale), composed of a protein capsid and nucleic acids.	Structurally complex (microscale), possessing complete cellular components (cell wall, flagella, LPS, etc.).
Immunogenicity	Moderate to high; effectively triggers antitumor adaptive immunity but is susceptible to rapid clearance by pre‐existing or induced neutralizing antibodies, limiting the efficacy of repeated systemic administration.	Highly potent; pathogen‐associated molecular patterns (PAMPs) act as natural adjuvants, robustly activating innate immunity and efficiently reversing the immunosuppressive microenvironment of “cold tumors.”
Targeting Mechanism	Receptor‐dependent: Relies on specific surface receptors for cellular entry; high specificity, but efficacy is restricted in tumors lacking target receptors.	Microenvironment‐dependent: Exploits unique conditions such as hypoxia, necrosis, and immunosuppression for colonization; independent of specific receptors and capable of reaching regions inaccessible to conventional drugs.
Genetic Manipulability	Small and compact genomes with limited exogenous gene payload capacity (typically <5–8 kb), which may interfere with viral replication and packaging.	Extensive toolbox for genetic engineering; facilitates complex engineering with massive payload capacities on the genome or plasmids (>4 Mb).
Tumor Penetration	Primarily dependent on passive diffusion; limited penetration within dense tumor stroma and poor accessibility to core hypoxic regions.	Active chemotaxis and motility: Utilizes structures like flagella to actively penetrate dense stroma and colonize necrotic/hypoxic cores, achieving a more uniform distribution.
Systemic Toxicity Risk	Generally low (following attenuation); systemic inflammatory responses are typically manageable.	Higher potential risk, including sepsis or cytokine storms; however, risks are effectively mitigated through attenuation (e.g., virulence gene knockout) and synthetic “suicide switches.”
Clinical and Regulatory Status	Relatively mature; several products are commercially available (e.g., T‐VEC, H101), supported by a well‐established clinical trial framework.	In a phase of rapid development; most candidates are in Phase I–III trials. Although clinical translation started later, progress is accelerating.
Administration Route	Predominantly intratumoral injection; systemic (intravenous) administration is often compromised by neutralizing antibodies and complement clearance, leading to off‐target effects.	Flexible administration; both intratumoral and intravenous routes are viable. Due to microenvironment targeting, they can enrich in multiple or metastatic lesions following systemic delivery, offering greater potential.
Production and Cost	Complex processes and high costs; requires eukaryotic systems (e.g., Vero cells, chick embryos) for cultivation, involving tedious downstream purification and large‐scale manufacturing challenges.	Established processes and low costs; allows for rapid, large‐scale expansion in simple fermenters (using LB/TB media), facilitating industrial mass production with significant cost advantages.
Safety and Controllability	Relies on inherent replication restrictions (e.g., tumor‐specific promoters) or antiviral drugs as safety backups.	Multi‐layered and more flexible regulatory strategies, including auxotrophy, inducible suicide systems, and quorum‐sensing switches, enabling dynamic clearance or self‐limitation post‐treatment.

As illustrated in Table 4, while oncolytic viruses, as pioneers of “living medicines,” have validated their clinical utility through commercially available products, oncolytic bacteria demonstrate unique advantages in addressing the profound challenges of solid tumor therapy. Unlike viruses, which primarily rely on intratumoral injection and struggle to penetrate dense stroma, oncolytic bacteria offer diverse administration routes, an innate capacity to actively target hypoxic cores, and extensive potential for synthetic biology‐based modification. These attributes provide a novel direction for treating deep‐seated tumor tissues and distant metastases.

Despite these significant advantages, no oncolytic bacteria products have been approved for clinical use to date. This is due to the fact that bacteria, as complex organisms with autonomous metabolism, present more unique challenges in regulatory and translational science than viruses. First, there is a discrepancy in historical precedent and biosafety concerns; viral vectors benefit from decades of success in vaccinology, whereas the approval pathways for bacterial therapies remain immature. Furthermore, the superior environmental resilience of bacteria imposes significant pressure on biosafety containment. Second, scientific and manufacturing hurdles persist; the expansive bacterial genome renders metabolic complexity and colonization dynamics within the tumor microenvironment difficult to predict. Simultaneously, the CMC required to maintain consistent potency in live bacterial products present high technical barriers. Additionally, systemic immunotoxicity is a critical factor; the potent immunostimulatory molecules (e.g., LPS, flagellin) carried by live bacteria can still trigger severe cytokine storms in humans, even in an attenuated state. We have systematically summarized these five critical barriers hindering the clinical translation of oncolytic bacteria in Table 5.

**TABLE 5:** Critical Barriers to the Clinical Translation of Oncolytic Bacteria.

Challenge Categories	Description	Impact
Historical Precedent	Viral vectors benefit from decades of successful application and established regulatory pathways in the fields of vaccinology and gene therapy.	Regulatory agencies lack a historical precedent for bacteria as “living medicines,” leading to a more cautious and stringent approval process.
Systemic Immunotoxicity	Live bacteria harbor potent immunostimulatory components (e.g., LPS, flagellin). Even with attenuated strains, intravenous administration may trigger a cytokine storm.	The resulting narrow therapeutic window represents the most significant safety obstacle to dose escalation and regulatory approval.
Biosafety and Environmental Persistence	Unlike viruses, which typically lose viability rapidly outside a host, bacteria—particularly spore‐forming species—exhibit exceptional environmental resilience.	Potential risks of environmental contamination through patient shedding and horizontal gene transfer (HGT) necessitate rigorous biosafety containment.
Metabolic Complexity	Bacteria possess expansive genomes and autonomous metabolic networks; their in vivo colonization dynamics are heavily influenced by host nutritional status.	The biological behavior of bacteria within the tumor microenvironment (e.g., growth kinetics and metabolite production) is far less predictable than that of structurally simpler viruses.
CMC Challenges	Ensuring the viability, potency, and batch‐to‐batch consistency of live bacterial products while minimizing impurities like endotoxins.	Industrial‐scale manufacturing is hindered by the current lack of standardized quality control frameworks.

In summary, although the regulatory approval of oncolytic bacteria currently faces multifaceted challenges, the rapid advancement of gene‐editing technologies and synthetic biology is systematically addressing these issues of safety and controllability. Oncolytic bacteria are steadily bridging the gap between laboratory research and clinical application, positioning them to become the next major breakthrough in the field of living therapeutics following oncolytic viruses.

### 6.3 Approved Bacterial Drugs Cases

Currently, most approved live bacterial drugs are primarily indicated for gastrointestinal disorders. For instance, Bifico, a formulation containing Bifidobacterium longum, Lactobacillus acidophilus, and Enterococcus faecalis, is primarily indicated for conditions associated with intestinal flora imbalance, including the prevention of necrotizing enterocolitis (NEC) in premature infants and the treatment of Helicobacter pylori‐related gastritis. It was approved as a prescription drug by China's Ministry of Health in April 1995. This approval process strictly complied with the drug approval regulations in effect at that time. Enterprises were required to submit comprehensive research data detailing the microbial taxonomic characteristics of each strain, including cell morphology and physiological and biochemical properties, as well as gene sequence analysis data to confirm the specificity and traceability of the strains. Additionally, multi‐batch stability studies were required to demonstrate that key indicators of the drug (e.g., viable bacterial count, activity, and genetic stability of the strains) met quality standards under various storage conditions. Regarding safety, animal experiments (e.g., long‐term toxicity tests and acute toxicity tests) were required to assess potential adverse reactions induced by the drug. Subsequently, in October 2002, it was approved as an over‐the‐counter (OTC) drug by China's regulatory authority, the FDA. This required enterprises to further demonstrate the safety and efficacy of the drug when used without the guidance of professional medical personnel, including conducting large‐scale clinical observations to collect adverse reaction data following patient self‐administration, thereby verifying its safety in broader population usage scenarios [159].

In production, manufacturing must be conducted in a GMP‐compliant facility. The strain culture step involves inoculating the original strains of Bifidobacterium longum, Lactobacillus acidophilus, and Enterococcus faecalis into appropriate media, respectively, followed by expansion culture in strictly controlled anaerobic or micro‐aerobic environments (e.g., Bifidobacterium longum requires an anaerobic environment, whereas Lactobacillus acidophilus requires a micro‐aerobic environment), with precise regulation of culture conditions such as temperature and pH. Once the strains reach a specific concentration, the bacteria are harvested via centrifugation, filtration, or other technical methods, and appropriate protective agents are added. The live bacteria are then processed into freeze‐dried powder using a vacuum freeze‐drying process to extend their shelf life and preserve activity. Finally, the freeze‐dried powder is uniformly mixed with other excipients and formulated into capsules or tablets (facilitating patient administration) using equipment such as capsule filling machines or tablet presses. Throughout the production process, strict quality control is required at each stage, including regular inspections of medium contamination, compliance with viable count standards, and whether the microbial limit of the finished product meets specifications. Other approved live bacterial drugs include Golden Bifid (containing Bifidobacterium longum, Lactobacillus bulgaricus, and Streptococcus thermophilus), primarily indicated for the treatment of diarrhea [160], and Changlekang (a combined live bacterial powder of Clostridium butyricum and Bifidobacterium), indicated for preventing secondary diarrhea in children with pneumonia.

REBYOTA^TM^ (RBX2660) and VOWST^TM^ (SER‐109) have been approved by the US FDA for the prevention of recurrent *Clostridium difficile* infection (rCDI) in adults [161]. REBYOTA^TM^  is a fecal microbial suspension administered via enema, which acts by restoring intestinal flora balance to prevent CDI recurrence [162]; VOWST^TM^, in contrast, is an oral fecal microbial spore preparation that inhibits *Clostridium difficile* growth through the colonization of beneficial bacteria. Its manufacturing process complies with GMP requirements, encompassing steps such as healthy donor screening, microbial composition standardization, quality control, and stability testing. The approval of these products is based on data from randomized double‐blind clinical trials demonstrating their efficacy and safety in preventing Rcdi [163]. For instance, in the ECOSPOR III trial, VOWST^TM^ significantly reduced relapse rates, with an 8‐week relapse‐free rate of 88%—significantly higher than the placebo group's 60% [164, 165].

With ongoing research advancements, live bacterial drugs have expanded into the treatment of other systemic diseases. For example, Akkermansia muciniphila (AKK bacteria) has shown promise: clinical studies have demonstrated its ability to improve insulin resistance in obese mice, reduce blood glucose levels, and decrease body weight via regulation of flora metabolism [166, 167]. LACTIN‐V is another notable live bacterial drug, primarily indicated for preventing recurrent bacterial vaginosis (BV) and recurrent urinary tract infections (rUTI) [168, 169, 170]. Furthermore, it has the potential to reduce HIV susceptibility [171], warranting further investigation. In a randomized, double‐blind, placebo‐controlled Phase 2b trial conducted by Cohen et al., 228 women were enrolled. After 12 weeks of assessment, the trial effectively demonstrated LACTIN‐V's efficacy and safety: the treatment group had a 30% recurrence rate, compared to 45% in the placebo group [168].

However, no genetically engineered bacteria have been approved for direct use in tumor therapy, although several products have entered clinical stages. For instance, SYNB1891 is an engineered *Escherichia coli* strain capable of activating the STING pathway and inducing immune responses in the hypoxic tumor microenvironment. It is indicated for the treatment of solid tumors or lymphomas and is currently in Phase 1 clinical trials [172]; Salmonella typhimurium VNP20009 has had its virulence genes (*purI* and *msbB*) knocked out, which reduces endotoxin activity and enhances tumor targeting. It is currently in Phase 1 trials for metastatic melanoma and renal cell carcinoma [173]; the attenuated Listeria vaccine for advanced cancer (ANZ‐100) and the mesothelin‐expressing attenuated Listeria vaccine (CRS‐207) have completed Phase 1 trials. These vaccines have been shown to be safe with standard administration and capable of inducing immune activation [174]; Intratumoral injection of Clostridium novyi (C. novyi‐NT) spores was used to treat a patient with advanced leiomyosarcoma, resulting in reduced intraosseous and periosseous tumors. This indicates that C. novyi‐NT can precisely eradicate tumor tissue, although further clinical trials in selected patient populations are still required [175].

The approval of bacterial therapies remains in an exploratory phase; however, progress across multiple products demonstrates that live bacterial drugs can meet regulatory requirements through rigorous strain screening, standardized manufacturing processes, and innovative clinical trial designs. Looking ahead, with advancements in attenuation strategies, improvements in detection technologies, and refinements in regulatory approval mechanisms, attenuated bacterial tumor therapies are expected to accelerate their path to market under a clear risk‐benefit framework. This will ultimately offer new treatment options for cancer patients (Table 6).

**TABLE 6:** Summary of Viable Drugs.

Drug	Bacteria	Function	Phase	References
Bifico	*Bifidobacterium longum*, *Lactobacillus acidophilus*, and *Enterococcus faecalis*	Mainly indicated for diseases related to intestinal flora imbalance, such as preventing necrotizing enterocolitis (NEC) in premature infants and treating Helicobacter pylori‐related gastritis.	Approved	[159]
Golden Bifid	*Bifidobacterium longum*, *Lactobacillus bulgaricus*, and *Streptococcus thermophilus*	Mainly used to treat diarrhea.	Approved	[160]
Changlekang	*Clostridium butyricum* and Bifidobacterium combined live bacterial powder	Used to prevent secondary diarrhea in children with pneumonia.	Approved	
REBYOTA, VOWST	Fecal microbiota	the prevention of recurrent *Clostridium difficile* infection (rCDI) in adults.	Approved	[161, 162, 163, 164, 165]
AKK bacteria	*Akkermansia muciniphila*	Assisted weight loss and anti‐obesity, improve blood glucose and insulin resistance, regulate blood lipid.	AKK bacteria have not been included in China's ‘List of Strains Permitted for Food Use’.	[166, 167]
LACTIN‐V	*Lactobacillus curvatus*	Primarily used to prevent the recurrence of bacterial vaginosis (BV) and recurrent urinary tract infections (rUTI). Additionally, it has the potential to reduce susceptibility to HIV.	It is in phase 2 clinical trials for BV and in phase 2/3 clinical trials for UTI.	[168, 169, 170, 171]
SYNB1891	*Escherichia coli* Nissle 1917	Used to treat solid tumors or lymphoma.	Phase 1 clinical trial stage.	[172]
	Salmonella typhimurium VNP20009	Used to treat metastatic melanoma and renal cell carcinoma.	Phase 1 clinical trial stage	[173]
ANZ‐100, CRS‐207	Listeria	Used to treat mesothelioma, lung cancer, pancreatic cancer or ovarian cancer.	Phase 1 trial completed.	[174]
	*Clostridium novyi*	Used to treat solid tumors such as smooth muscle sarcoma.	Phase 1 clinical trial stage	[175]
BCG	*Mycobacterium bovis*	Prevent tuberculosis and treat bladder cancer.	Approved	[13, 14]

## 7 Conclusion

Attenuated bacteria‐mediated therapy leverages bacterial chemotaxis and immunomodulation for cancer treatment, yet clinical translation faces critical scientific and technical bottlenecks. Systematic optimization of safety, efficacy, and predictability is essential for its medical application. In clinical practice, balancing pathogenicity and anti‐tumor efficacy is paramount. This necessitates transitioning from random mutagenesis to precision gene editing—such as targeted knockouts of virulence factors and the integration of environment‐responsive gene switches—to achieve precise control over bacterial activity.

Although VNP20009 exhibits favorable safety profiles in immunocompetent hosts, it may still induce severe systemic infections in immunodeficient or immunocompromised individuals. The clinical outcomes of VNP20009—characterized by inconsistent colonization efficiency and potential safety risks in immunocompromised hosts—underscore the inherent difficulty in predicting and controlling bacterial behavior within the heterogeneous human tumor microenvironment and diverse immune landscapes, even when based on rational genetic engineering. These findings do not represent a failure of the design philosophy itself; rather, they epitomize the universal challenges associated with the translational gap between laboratory models and clinical application. Indeed, it is the pioneering nature of VNP20009's clinical trials that has established the strain as an invaluable ‘reference benchmark’ in the field. Given its well‐defined genetic background, extensive safety profile, and validated tumor tropism, VNP20009 provides a robust baseline for comparative studies [176]. Consequently, it continues to be widely cited as a gold standard in current oncotherapeutic research [177].

Artificial intelligence (AI) and machine learning are significantly accelerating the clinical translation of attenuated bacterial cancer therapies by addressing critical bottlenecks, such as the complexity of genetic circuit design, the unpredictability of in vivo behavior, and patient heterogeneity. Traditional genetic circuit design relies on experimental iterations that are time‐consuming, costly, and difficult to simultaneously optimize for quorum‐sensing thresholds, promoter strength, and metabolic burden, often resulting in leakage or insufficient expression. Deep learning algorithms, trained on massive datasets, can process sequence data and chassis parameters to directly predict steady‐state expression, oscillation periods, and growth inhibition. In practice, models can virtually screen thousands of candidates within hours, requiring only minimal validation to lock in optimal designs. This has shortened optimization cycles from months to weeks and significantly reduced failure rates [178].

Furthermore, the in vivo distribution kinetics of bacteria are profoundly influenced by the heterogeneity of the tumor microenvironment, making it difficult to extrapolate human clinical outcomes directly from small animal models. Machine learning integrates tumor imaging, immunological parameters, and bacterial physiological data. By employing supervised learning on preclinical datasets, these models can provide real‐time outputs of spatiotemporal distribution, peak concentrations, and clearance curves. This enables precise adjustments to dosage, administration timing, and combination regimens, thereby mitigating the risk of preclinical variability [179, 180]. Moreover, patient heterogeneity leads to substantial variations in the efficacy of “universal” strains. AI frameworks can integrate multi‐omics data—including tumor transcriptomics, proteomics, metabolomics, and microbiomics—to identify high‐response biomarkers (e.g., high STING activity or low Treg levels). This directly guides strain customization (e.g., selecting promoters that match a specific patient's oxygen gradient), facilitating the leap from universal treatments to precision medicine [181, 182].

In summary, AI and machine learning not only provide powerful computational support for the precise design and kinetic prediction of engineered bacteria but also demonstrate immense potential in overcoming clinical translation challenges and addressing individual patient differences. This data‐driven research paradigm is shifting bacterial therapy from traditional empirical exploration toward rationalized design. Through the deep integration of multi‐dimensional data, this approach significantly enhances therapeutic controllability and clinical response rates, laying a solid foundation for the development of next‐generation intelligent and personalized bacterial medicines.

Conventional in vitro methods fail to reflect complex in vivo dynamics. Precise therapy requires advanced visualization—synergizing optical, ultrasound, and MRI technologies—to evaluate treatment responses and guide genetic material release medicine. As living therapeutics, establishing automated, GMP‐compliant production is essential to ensure batch‐to‐batch uniformity and genetic stability. Furthermore, standardized approval pathways, multi‐center clinical databases, and long‐term safety monitoring are critical to move this therapy from the laboratory to the clinic, ultimately improving patient prognosis.

In summary, the clinical translation of attenuated bacterial tumor therapy is a systematic project encompassing basic research, technological development, production standards, and regulatory systems. Future efforts should focus on addressing key issues, including safety optimization, efficacy regulation, monitoring technologies, production specifications, and approval standards, through multidisciplinary integration and coordinated advancement of scientific research and regulatory frameworks. This will facilitate the translation of the therapy from the laboratory to the clinic, offering new treatment options for cancer and ultimately improving patients’ quality of life and prognosis.

## Funding

This research was supported by the National Natural Science Foundation of China (82473333), Jiangsu Province Social Development Projects (BE2023689), Construction Project of High Level Hospital of Jiangsu Province (LCZX202501), XZHMU‐QL Joint Research Fund (QL‐YB032), the Project of Invigorating Health Care through Science, Technology and Education (CXZX202234), the Basic Science (Natural Science) Research Project of the Jiangsu Higher Education Institutions (25KJB350010), the Medical Research Project of the Jiangsu Commission of Health (ZQ2025011) and the Scientific Research Startup Fund for Outstanding Talents of Xuzhou Medical University (RC20552411).

## Copyright Permissions

Relevant figures in this review are adapted or reproduced from previously published works with permission from the respective publishers or under applicable licenses.

## Conflicts of Interest

The authors declare no conflicts of interest.
